# Protein Restriction Effects on Healthspan and Lifespan in *Drosophila melanogaster* Are Additive With a Longevity-Promoting Diet

**DOI:** 10.1093/gerona/glad225

**Published:** 2023-09-22

**Authors:** Wei Zhang, Yunshuang Ye, Yinan Sun, Yongxuan Li, Mingxia Ge, Kangning Chen, Liping Yang, Guijun Chen, Jumin Zhou

**Affiliations:** Key Laboratory of Animal Models and Human Disease Mechanisms of Chinese Academy of Sciences/Key Laboratory of Healthy Aging Research of Yunnan Province, Kunming Key Laboratory of Healthy Aging Study, Kunming Institute of Zoology, Kunming, Yunnan, China; Kunming College of Life Science, University of Chinese Academy of Sciences, Beijing, China; Key Laboratory of Animal Models and Human Disease Mechanisms of Chinese Academy of Sciences/Key Laboratory of Healthy Aging Research of Yunnan Province, Kunming Key Laboratory of Healthy Aging Study, Kunming Institute of Zoology, Kunming, Yunnan, China; Key Laboratory of Animal Models and Human Disease Mechanisms of Chinese Academy of Sciences/Key Laboratory of Healthy Aging Research of Yunnan Province, Kunming Key Laboratory of Healthy Aging Study, Kunming Institute of Zoology, Kunming, Yunnan, China; Kunming College of Life Science, University of Chinese Academy of Sciences, Beijing, China; Key Laboratory of Animal Models and Human Disease Mechanisms of Chinese Academy of Sciences/Key Laboratory of Healthy Aging Research of Yunnan Province, Kunming Key Laboratory of Healthy Aging Study, Kunming Institute of Zoology, Kunming, Yunnan, China; Kunming College of Life Science, University of Chinese Academy of Sciences, Beijing, China; State Key Laboratory of Genetic Resources and Evolution/Key Laboratory of Healthy Aging Research of Yunnan Province, Kunming Institute of Zoology, Chinese Academy of Sciences, Kunming, Yunnan, China; Key Laboratory of Animal Models and Human Disease Mechanisms of Chinese Academy of Sciences/Key Laboratory of Healthy Aging Research of Yunnan Province, Kunming Key Laboratory of Healthy Aging Study, Kunming Institute of Zoology, Kunming, Yunnan, China; Kunming College of Life Science, University of Chinese Academy of Sciences, Beijing, China; Key Laboratory of Animal Models and Human Disease Mechanisms of Chinese Academy of Sciences/Key Laboratory of Healthy Aging Research of Yunnan Province, Kunming Key Laboratory of Healthy Aging Study, Kunming Institute of Zoology, Kunming, Yunnan, China; Key Laboratory of Animal Models and Human Disease Mechanisms of Chinese Academy of Sciences/Key Laboratory of Healthy Aging Research of Yunnan Province, Kunming Key Laboratory of Healthy Aging Study, Kunming Institute of Zoology, Kunming, Yunnan, China; Key Laboratory of Animal Models and Human Disease Mechanisms of Chinese Academy of Sciences/Key Laboratory of Healthy Aging Research of Yunnan Province, Kunming Key Laboratory of Healthy Aging Study, Kunming Institute of Zoology, Kunming, Yunnan, China; KIZ/CUHK Joint Laboratory of Bioresources and Molecular Research in Common Diseases, Kunming, Yunnan, China; (Biological Sciences Section)

**Keywords:** Aging, Immune senescence, Longevity

## Abstract

Aging of the organism is associated diminished response to external stimuli including weakened immune function, resulting in diseases that impair health and lifespan. Several dietary restriction modalities have been reported to improve health and lifespan in different animal models, but it is unknown whether any of the lifespan-extending dietary treatments could be combined to achieve an additive effect. Here, we investigated the effects of halving amino acids components in the HUNTaa diet, a synthetic medium known to extend lifespan in *Drosophila*. We found that dietary restriction by halving the entire amino acid components (DR group) could further extend lifespan and improve resistance to oxidative stress, desiccation stress, and starvation than flies on HUNTaa diet alone (wt group). Transcriptome analysis of *Drosophila* at 40, 60, and 80 days of age revealed that genes related to cell proliferation and metabolism decreased with age in the wt group, whereas background stimulus response and amino acid metabolism increased with age. However, these trends differed in the DR group, that is, the DR flies had downregulated stress response genes, including reduced background immune activation. Infection experiments demonstrated that these flies survived longer after feeding infection with *Serratia marcescens* and *Enterococcus faecalis*, suggesting that these flies had stronger immune function, and therefore reduced immune senescence. These results demonstrated that halving the entire amino acid components in the HUNTaa diet further extended health and lifespan and suggested that lifespan-extending diet and dietary restriction treatment could be combined to achieve additive beneficial results.

Dietary restriction is a broad term describing the reduction in specific dietary components or in amounts of food provided, which has been shown to extend lifespan and improve healthspan in a wide range of animals including *yeast*, *Caenorhabditis elegans*, *Drosophila*, and mouse (1). Dietary restriction includes reduction in total calorie intake (Caloric restriction) (2), reduction in protein content of the diet (Protein restriction) (3), reduction in the daily period of food intake (Time-restricted feeding) (4), short-term daily or weekly fasting periods of 12–48 hours (Intermittent fasting) (5), and any diet compositions or feeding regimen designed to enhance healthy longevity (6–8). Dietary restriction enhances stem cell and mitochondrial function, strengthens autophagy, tissue repair, reduces cell senescence, and inflammation, and thus improves stress resistance to achieve a long and healthy life through upregulation of insulin sensitivity, SIRT, AMPK, DNA repair, proteostasis or downregulation of the mTOR pathway, glucose metabolism, lipid metabolism, and amino acid metabolism (9–16).

Dietary restriction experiments in *Drosophila* usually include changes in food composition, caloric restriction, and intermittent fasting (17). Earlier studies found that *Drosophila* lifespan could be prolonged by altering the availability of live yeast in the medium or diluting the medium (18,19), and that female *Drosophila* were more sensitive to dietary restriction than males (20).

Dietary restriction in *Drosophila* affects processes such as the insulin/IGF-like signaling pathway, nutrient-sensitive pathways, glucose metabolism, lipid metabolism, and amino acid metabolism. The *Drosophila* insulin receptor substrates Chico, AKT, and dFOXO are all important factors in regulating *Drosophila* longevity signaling pathway (21), and reducing the growth signaling pathway improves *Drosophila* survival (22). Amino acid-restricted dietary structures have also been shown to extend lifespan and improve health in different species. In *Drosophila*, restriction of methionine, branched-chain amino acids, leucine, isoleucine, or valine can extend the lifespan of flies (23–26), but the molecular mechanisms are not well understood.

However, not all nutritional restrictions are beneficial for the organism. When protein content is either too low or too high in the diet, it can shorten lifespan and reduce stress resistance in *Drosophila* (27). Chemically defined cultures HUNTaa and Yaa food could both prolong the lifespan of *Drosophila* (28), although the molecular mechanism is not clear. Unexpectedly, the Yaa diet shortens the lifespan when there is a reduction of either one of the essential amino acids (29). This raise a question whether and how lifespan extension diet treatments could be combined to have additive beneficial effects. To answer this question, we studied the effect of halving amino acid components on the already known lifespan-extending synthetic HUNTaa medium.

We found that halving amino acid component of the HUNTaa diet (DR group) from 30 days of age on improved resistance to oxidative stress, desiccation stress, starvation stress, and infection to extend lifespan. Transcriptome analyses revealed the DR group had downregulated stimulatory response and immune process genes compared to the wt group (flies feed with HUNTaa). The downregulation indicated an improved immunity, and this was confirmed by infection assays in these flies. These results demonstrated that further improvements in health and lifespan extension in *Drosophila* can be achieved by adjusting the amino acid ratios in the HUNTaa diet formula that already extends lifespan in *Drosophila*, and suggested that health and lifespan-extending factors could be combined to achieve an additive effect.

## Results

### Dietary Restriction by Halving Amino Acid Components in the HUNTaa Food Extends Lifespan and Improves Health in *Drosophila*

**Figure 1.**
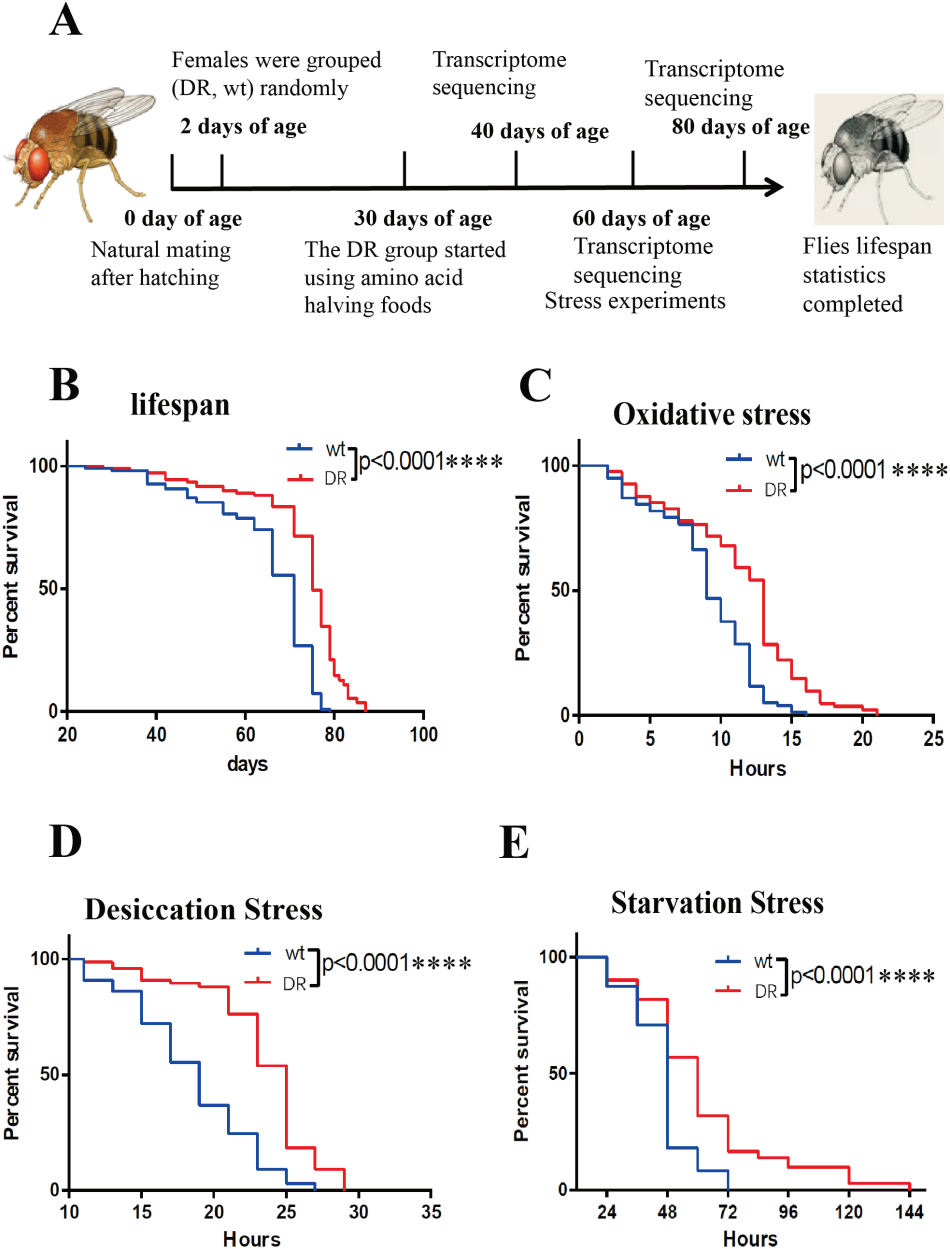
Survival and stress survival of DR and wt groups. (A) Summary of the experimental process. (B) Survival of DR and wt groups, each group used flies *n* = 109, log-rank test, *p* < .0001(****). (C) Survival of 30% hydrogen peroxide-treated DR and wt groups, each group used flies *n* > 75, log-rank test, *p* < .0001(****). (D) Survival of DR and wt groups at desiccation stress, each group used flies *n* > 65, log-rank test, *p* < .0001(****). (E) Survival of DR and wt groups at starvation stress, each group used flies *n* = 72, log-rank test, *p* < .0001(****).

To investigate the effects of halving amino acid component of the HUNTaa diet on *Drosophila* lifespan and health, we did lifespan analysis, stress tests, and transcriptome analysis. We first let *Drosophila* adults’ mate freely for the first 2 days of age, then female *Drosophila* were randomly divided into wt and DR groups (all *Drosophila* were on HUNTaa food (28)); the DR group started to change to halving amino acid component from the HUNTaa food at 30 days of age. These flies were also subject to stress test for healthspan analyses, including oxidative stress, starvation stress, and desiccation stress experiments performed at 60 days of age (Figure 1A, Table 1).

**Table 1.** Food Composition of DR and wt Groups

	Ingredient	Stock	Per liter (wt group)	Per liter (DR group)	Manufacturer, Example Order Number
Gelling agent	Agar		20 g	20 g	Sigma, A7002
Base	Buffer	10×: 30 mL/L glacial acetic acid, 30 g/L KH_2_PO_4_, 10 g/L NaHCO_3_	100 mL	100 mL	Fisher, A/0400/PB15 Sigma, P9791 Sigma, S8875
Sugar	Sucrose		17.12 g	17.12 g	Sangon Biotech, S0335
Amino acids	l-isoleucine, l-leucine, l-tyrosine		1.82 g, 1.21 g, 0.42 g	0.91 g, 0.605 g, 0.21 g	Sigma, I2752 Sigma, L8912 Sigma, T3754
Metal ions	CaCl_2_·6H_2_O	1000×: 250 g/L	1 mL	1 mL	Sigma, C7902
	CuSO_4_·5H_2_O	1000×: 2.5 g/L	1 mL	1 mL	Sigma, C7631
	FeSO_4_·7H_2_O	1000×: 25 g/L	1 mL	1 mL	Sigma, F7002
	MgSO_4_	1000×: 250 g/L	1 mL	1 mL	Sigma, M7506
	MnCl_2_·4H_2_O	1000×: 1 g/L	1 mL	1 mL	Sigma, M3634
	ZnSO_4_·7H_2_O	1000×: 25 g/L	1 mL	1 mL	Sigma, Z0251
Cholesterol	Cholesterol	20 mg/mL in EtOH	15 mL	15 mL	Sigma, C8667
Amino acids	Essential amino acid stock solution	8 g/L l-arginine; 10 g/L l-histidine; 19 g/L l-lysine (HCl); 8 g/L l-methionine; 13 g/L l-phenylalanine; 20 g/L l-threonine; 5 g/L l-tryptophan; 28 g/L l-valine	60.51 mL	30.255 mL	Sigma, A5131; Sigma, H8000; Sigma, L5626; Sigma, M9625; Sigma, P2126; Sigma, T8625; Sigma, T0254; Sigma, V0500
Amino acids	Nonessential amino acid stock solution	35 g/L l-alanine; 17 g/L l-asparagine; 17 g/L l-aspartic acid; 1 g/L l-cysteine HCl; 25 g/L l-glutamine; 32 g/L glycine; 15 g/L l-proline	60.51 mL	30.255 mL	Sigma, A7627; Sigma, A0884; Sigma, A6683; Sigma, C1276; Sigma, G3126; Sigma, G7126; Sigma, P0380
	Sodium glutamate stock solution	19 g/L l-serine; 100 g/L sodium glutamate	15.13 mL	7.57 mL	Sigma, S4500; Sigma, G5889
Vitamins	Vitamin solution	125×: 0.1 g/L thiamine (aneurin); 0.05 g/L riboflavin; 0.6 g/L nicotinic acid; 0.775 g/L Ca pantothenate; 0.125 g/L pyridoxine (HCl); 0.01 g/L biotin	21 mL	21 mL	Sigma, T4625; Sigma, R4500; Sigma, N4126; Sigma, P2250; Sigma, P9755; Sigma, B4501
Other nutrients		125×: 6.25 g/L choline chloride; 0.63 g/L myoinositol; 8.13 g/L inosine; 7.5 g/L uridine	8 mL	8 mL	Sigma, C1879; Sigma, I7508; Sigma, I4125; Sigma, U3750
Preservatives	Nipagin; propionic acid	100 g/L methyl4-hydroxybenzoate in 95% EtOH	15 mL; 6 mL	15 mL; 6 mL	Clariant Nipagin M; Sigma, P5561
	Water (milliQ)		1 L minus combined volume of additions to be	1 L minus combined volume of additions to be	

We found that the DR group lived longer than the wt group, the median survival time increased from 71 to 74 days, an increase of 4.2% (Figure 1B). This result confirmed that the dietary restriction of halving amino acid component of the HUNTaa diet extended lifespan than HUNTaa food in flies.

#### Oxidative stress test

To test the ability of the wt and DR groups to resist oxidative stress, we added 30% hydrogen peroxide and 6% glucose as the medium for survival statistics. The results showed that the resistance to oxidative stress was improved in the DR group, the median survival time increased from 9 to 13 hours, an increase of 44.4% (Figure 1C).

#### Desiccation stress test

During desiccation stress, flies were subjected to desiccation stress at 25°C by incubating in vials without water and food. DR group also had increased desiccation resistance, with the median survival extended from 19 to 25 hours, an increase of 31.6% (Figure 1D).

#### Starvation stress test

DR and wt groups of *Drosophila* were subjected to starvation stress by incubating them in medium with only water and agar. As expected, the DR group again lived longer than wt group, the median survival time increased from 48 to 60 hours, an increase of 25% (Figure 1E).

In conclusion, those data suggest that the dietary restriction not only can extends flies’ lifespan but also can improve the ability of resistance to oxidative, desiccation, and starvation stress.

### The DR Group Alters the Expression Pattern of Gene Clusters That Change With Age in the wt Group

To uncover how the expression pattern of gene clusters changes in wt and DR groups during aging. We performed transcriptome sequencing in 2 groups of *Drosophila* at 40, 60, and 80 days of age. First, in the wt group by limma (*p* < .05) for multiple time point difference analysis, we found 623 differentially expressed genes during aging, then we performed time-series analysis of the expression profiles of differential genes across 40 to 80 days of age using the Mfuzz R package.

**Figure 2.**
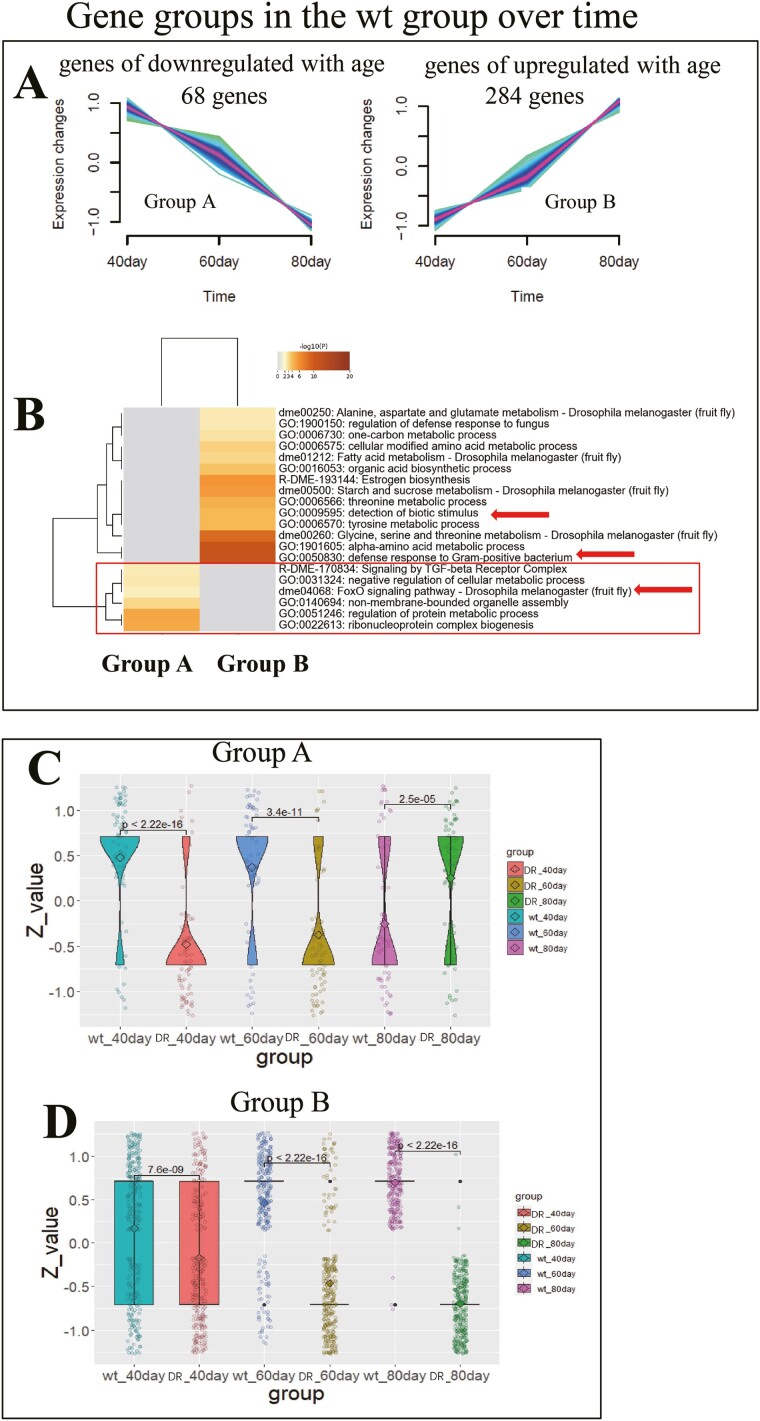
Genes of upregulated and downregulated in wt group during aging. (A) Gene groups in the wt group over time. Two groups (Group A and Group B) were obtained from 622 genes that change with age in the wt group (limma, *p* < .05) based on Euclidean distance and *c*-means objective function (22), 68 genes of downregulated with age in Group A, 284 genes of upregulated with age in Group B. (B) Enrichment analysis of Group A and Group B list was conducted using Metascape. Pathway terms with *p* < .01, minimum gene count of 3, and enrichment factor >1.5 were retained. (C) The *Z* value of Group A genes in the DR and wt groups, performed *t* test at 40, 60, and 80 days of age, respectively. DR lower than wt group at 40 days of age, *p* < 2.22e-16(****); DR lower than wt group at 60 days of age, *p* = 3.4e-11(****); DR lower than wt group at 80 days of age, *p* = 2.5e-05(****). (D) The *Z* value of Group B genes in the DR and wt groups, performed *t* test at 40, 60, and 80 days of age, respectively. DR lower than wt group at 40 days of age, *p* = 7.6e-09(****); DR lower than wt group at 60 days of age, *p* < 2.22e-16(****); DR higher than wt group at 80 days of age, *p* < 2.22e-16(****).

We found that 68 genes related to FoxO pathway and cellular metabolism decreased during aging in Group A (Figure 2A and B), and 284 genes related to stimulus response and amino acid metabolism increased during aging in Group B (Figure 2A and B). All genes enriched in Group A and Group B in Supplementary Table 1.

In order to reveal how the DR group affects the expression of Group A and Group B genes during the aging process. We took the *Z* value of these genes in the DR and wt groups, and then performed *t* tests at 40, 60, and 80 days of age, respectively. Interestingly, the expression of genes in Group A of DR group was lower than that in wt group at 40 and 60 days of age, and higher than that in wt group at 80 days of age (Figure 2C). The expression of genes in Group B of DR group was lower than wt group at 40, 60, and 80 days of age (Figure 2D).

This suggests that the dietary restriction can delay the effects of *Drosophila* on cellular metabolism and the FoxO pathway in natural aging, which is beneficial for life and health.

### The DR Group Reduces Stimulus Response and Upregulates Ribosomal Biological Processes

**Figure 3.**
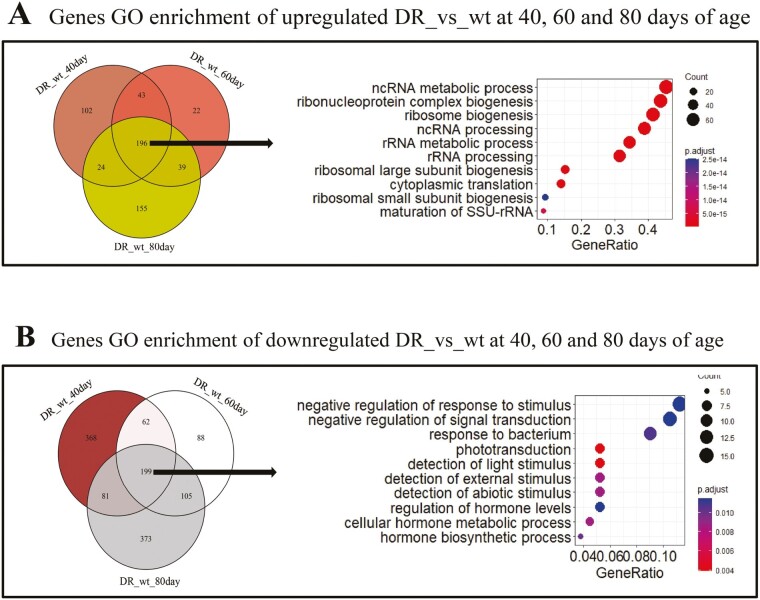
Functional enrichment of genes differentially expressed in the DR group compared to the wt group at 40, 60, and 80 days of age. (A) Functional enrichment of genes upregulated in the DR group compared to the wt group at 40, 60, and 80 days of age. Top 10 biological processes of Gene ontology terms (GO_enrichment) are shown. (B) Functional enrichment of genes downregulated in the DR group compared to the wt group at 40, 60, and 80 days of age. Top 10 biological processes of Gene ontology terms (GO_enrichment) are shown.

To reveal the effect of halving amino acid on biological processes in the HUNTaa diet, we performed gene differential expression analysis between DR and wt groups using limma at 40, 60, and 80 days of age (BH-corrected *p* < .05), respectively. The genes that were differentially up- and downregulated at each of the 3 age points were intersected to obtain differential genes ages in the DR compared to the wt group, and then the intersected genes were enriched for Gene Ontology (GO). The results showed that there were 196 differentially upregulated crossover genes, which are enriched in rRNA metabolism, ribosomal biological processes, and RNA transcription (Figure 3A); 199 differentially downregulated crossover genes were also found, which are enriched in multiple stimulus response processes (Figure 3B). These suggest that the effects of the dietary restriction are upregulating processes related to cellular metabolism, and downregulating genes related to stimulus response processes, and that these effects did not vary during aging.

### The DR Group Reduces the Immune Response in Natural Aging

Stimulus response processes include immune response, protein phosphorylation, cell activation, response to stress, and cell migration. During aging, flies with extended lifespan tend to downregulate the expression of immune effector genes, such as antimicrobial peptides (AMPs). In contrast, upregulation of immune pathways in *Drosophila* is a sign of immune senescence, which decreases survival in the event of infection (30).

**Figure 4.**
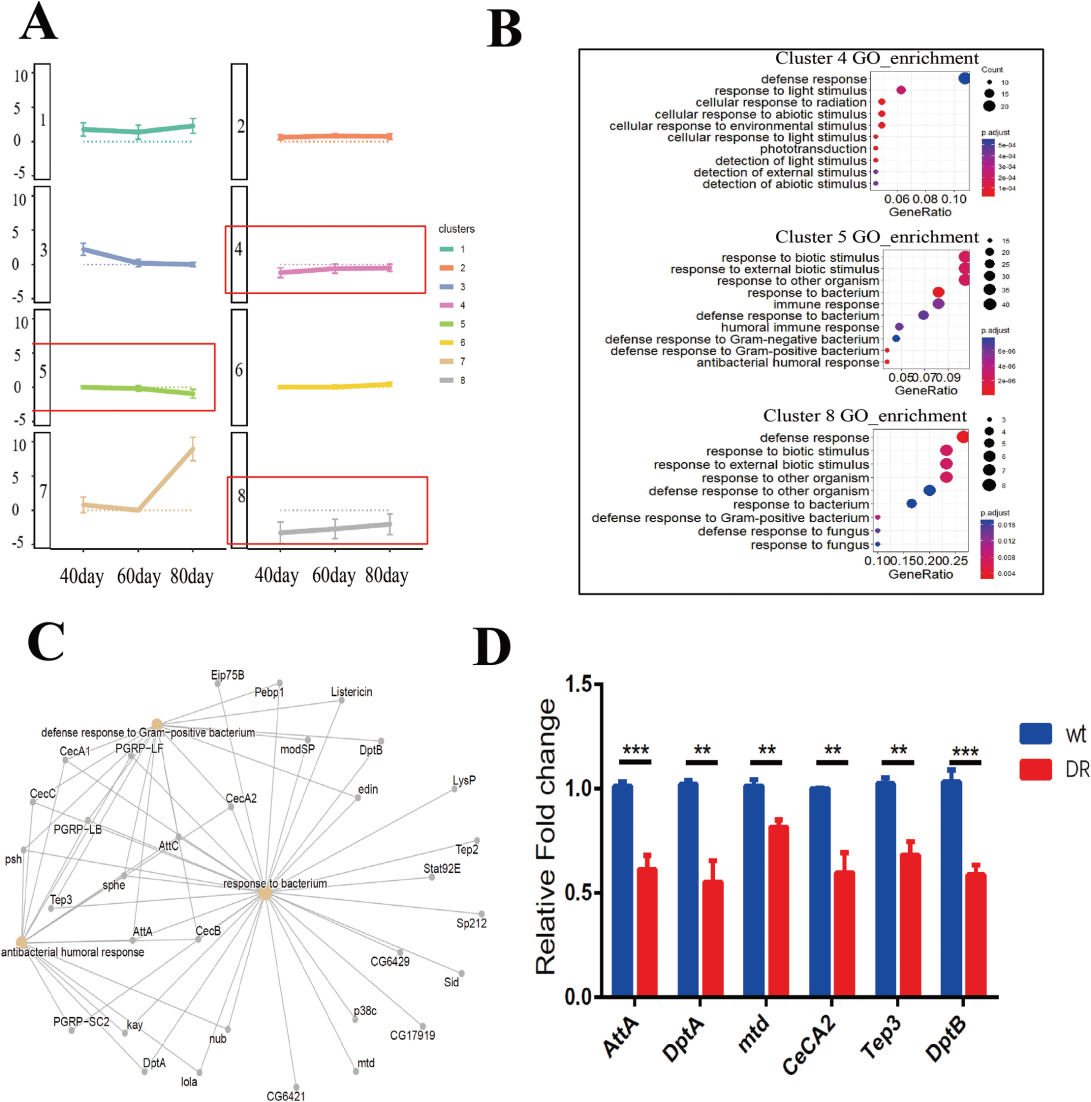
Clustering of gene clusters based on the differential multiplicity of genes in the DR group compared to the wt group at 40, 60, and 80 days of age. (A) Eight gene clusters were obtained from the Log2FoldChange of DR group compared to the wt group differential expression genes at 40, 60, and 80 days of age, data are shown as mean ± SEM (39). (B) Top 10 biological processes of Gene ontology terms (GO_enrichment) of cluster 4, cluster 5, and cluster 8 are shown. (C) Cnetplot of 3 biological processes in Cluster 5 are shown. (D) Transcript levels of response to bacterium genes AttA, DptA, mtd, CeCA2, Tep3, DptB of DR group and wt group at 60 days of age in Drosophila by qPCR, *n* = 3, 2-tailed *t* test, *p* < .001(***), *p* < .01(**), *p* < .01(**), *p* *<* .01(**), *p* < .01(**), *p* < .001(***).

To further understand whether the prolonged lifespan of the DR group was associated with a decrease in stimulus response-related genes. We performed gene differential expression analysis (BH-corrected *p* < .05) for the DR and wt groups at 40, 60, and 80 days of age, respectively, using the DESeq2 R package, and performed gene clustering based on the log_2_FoldChange values of these differential genes. We obtained 8 gene clusters (clusters 1–8) with the number of genes in each gene cluster: 38, 312, 112, 323, 519, 919, 20, 49 (Supplementary Table 2). Cluster 3 is differentially upregulated genes at age of 40 days, cluster 6 differentially upregulated genes at age of 80 day, and cluster 7 is differentially upregulated genes at both age of 40 and 80 days, and with a higher upregulation at 80 days (Figure 4A). Importantly, clusters 1,2 upregulated at all 3 ages and similar in magnitude compared to the wt group (Figure 4A). Clusters 4,8 are downregulated at all 3 ages compared to the wt group and cluster 5 is differentially downregulated genes at age of 80 days. Interestingly, the functions of clusters 4,5,8 are mainly related to the immune response and cell stimulation response processes (Figure 4A–C). The levels of response to bacterium-related gene transcripts were lower in the DR group than in the wt group at 60 days of age (Figure 4D), suggesting the possibility of improved immune function in the DR group due to reduced background levels of immune gene expression (Figure 4B and D) (31).

### The DR Group Improves the Immune System Function of *Drosophila* in Aging

To verify whether the DR group improves immune function in *Drosophila*. We performed feeding infections with gram-negative (*Serratia marcescens*, DAP-type) and positive (*Enterococcus faecalis*, Lys-type) bacteria to 60-day-old flies. As control, we measured bacterial proliferation in the medium and *Drosophila* feeding amount in the 2 diet groups for 24 hours (Supplementary Figures 1 and 2) and found no differences between the two. This result suggested that the fed amount of bacteria are not significantly different between the wt and DR groups of flies. Similarly, no difference in bacterial load outside the oral diet of *Drosophila* after 24 and 48 hours of bacterial infection was found (Supplementary Figure 3). Therefore, we can assume that there was no difference in oral bacterial load between the 2 groups of *Drosophila* at 24 hours. Based on this, we conducted experiments to count the survival rate of *Drosophila* and to detect the bacterial load and expression of AMPs in *Drosophila* at 48 hours by changing the oral bacterial solution every 24 hours.

**Figure 5.**
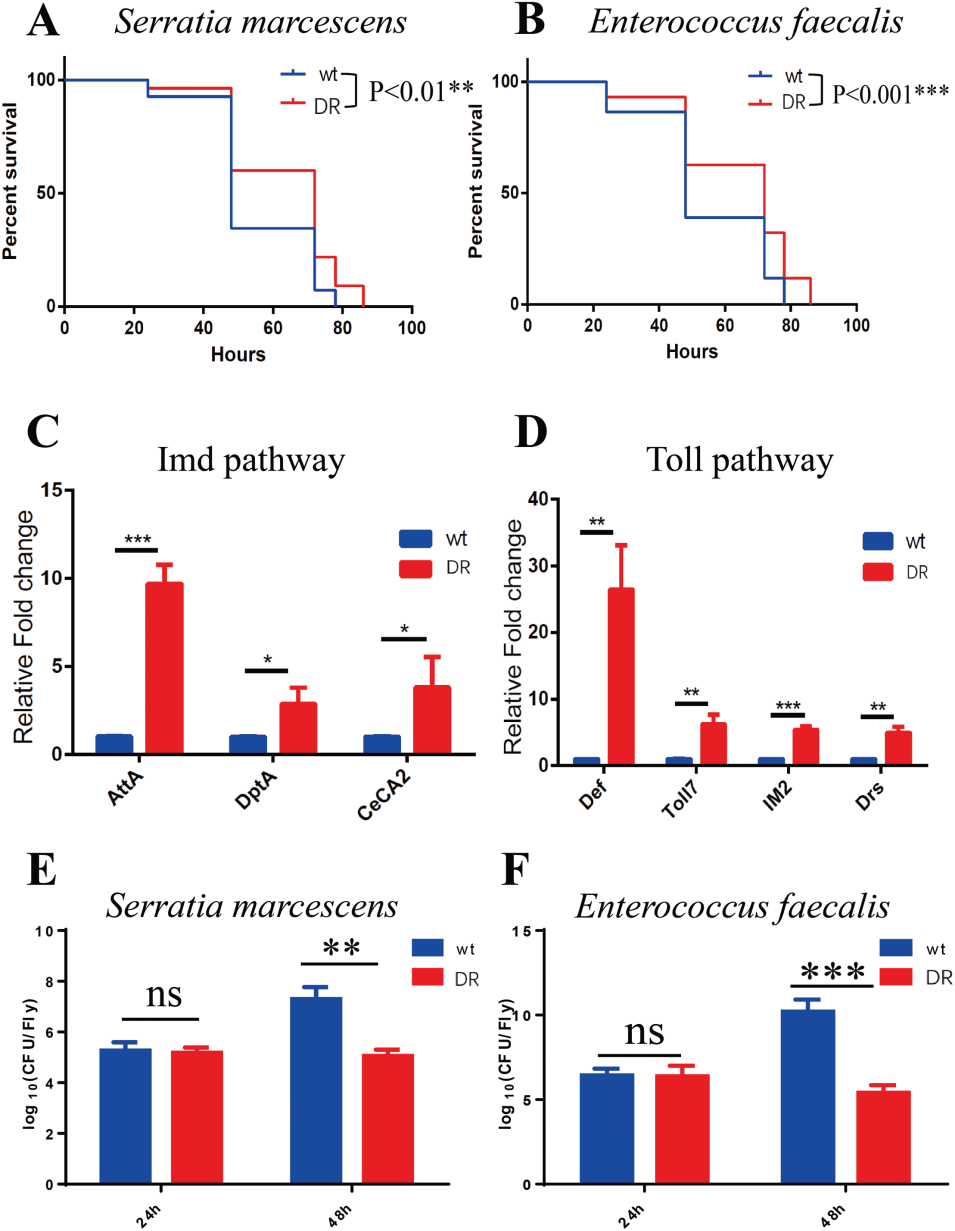
Survival of *Serratia marcescens* and *Enterococcus faecalis* infection in wt and DR groups and detection of transcript levels of antimicrobial peptides of the corresponding activated pathways. (A) Survival of DR and wt groups after *Serratia marcescens* infected, each group used flies *n* > 55, log-rank test, *p* < .01(**). (B) Survival of DR and wt groups after *Enterococcus faecalis* infected, each group used flies *n* > 55, log-rank test, *p* < .001(***). (C) Transcript levels of Imd pathway genes (AttA, DptA, CeCA2) in the DR and wt groups were measured by qPCR 48 hours after infection with *Serratia marcescens*, *n* = 3, 2-tailed *t* test, *p* < .001(***), *p* < .05(*), *p* < .05(*). (D) Transcript levels of Toll pathway genes (Def, Toll7, IM2, Drs) in the DR and wt groups were measured by qPCR 48 hours after infection with *Enterococcus faecalis*, *n* = 3, 2-tailed *t* test, *p* < .01(**), *p* < .01(**), *p* < .001(***), *p* < .01(**). (E) Determination of CFU/fly after 24 and 48 hours of *Serratia marcescens* infection in wt and DR groups, *n* = 3, 2-tailed *t* test, 24 hours *p* > .05(ns), 48 hours *p* < .01(**).

We found that DR group lived longer than wt group after both *S marcescens* and *E faecalis* infections, the median survival time both increased from 48 to 72 hours, an increase of 50% (Figure 5A and B). The DR group showed increased expression of antimicrobial peptide genes downstream of the Imd (*AttA*, *DptA*, *CeCA2*) and Toll (*Def*, *Toll7*, *IM2*, *Drs*) pathways in *Drosophila* 48 hours after infection with *S marcescens* and *E faecalis* (Figure 5C and D), indicating that the DR group had greater resistance to bacterial infection. The bacterial colony forming units in the DR group of *Drosophila* were not different from that of the wt group at 24 hours after infection with *S marcescens* or *E faecalis*, and were lower than that of the wt group at 48 hours (Figure 5E and F), indicating that the ability of *Drosophila* in the DR group to clear the bacteria at 48 hours was stronger than that of the wt group. These results demonstrated that the dietary restriction improves immune function in middle- and old-aged *Drosophila*.

## Discussion

**Figure 6.**
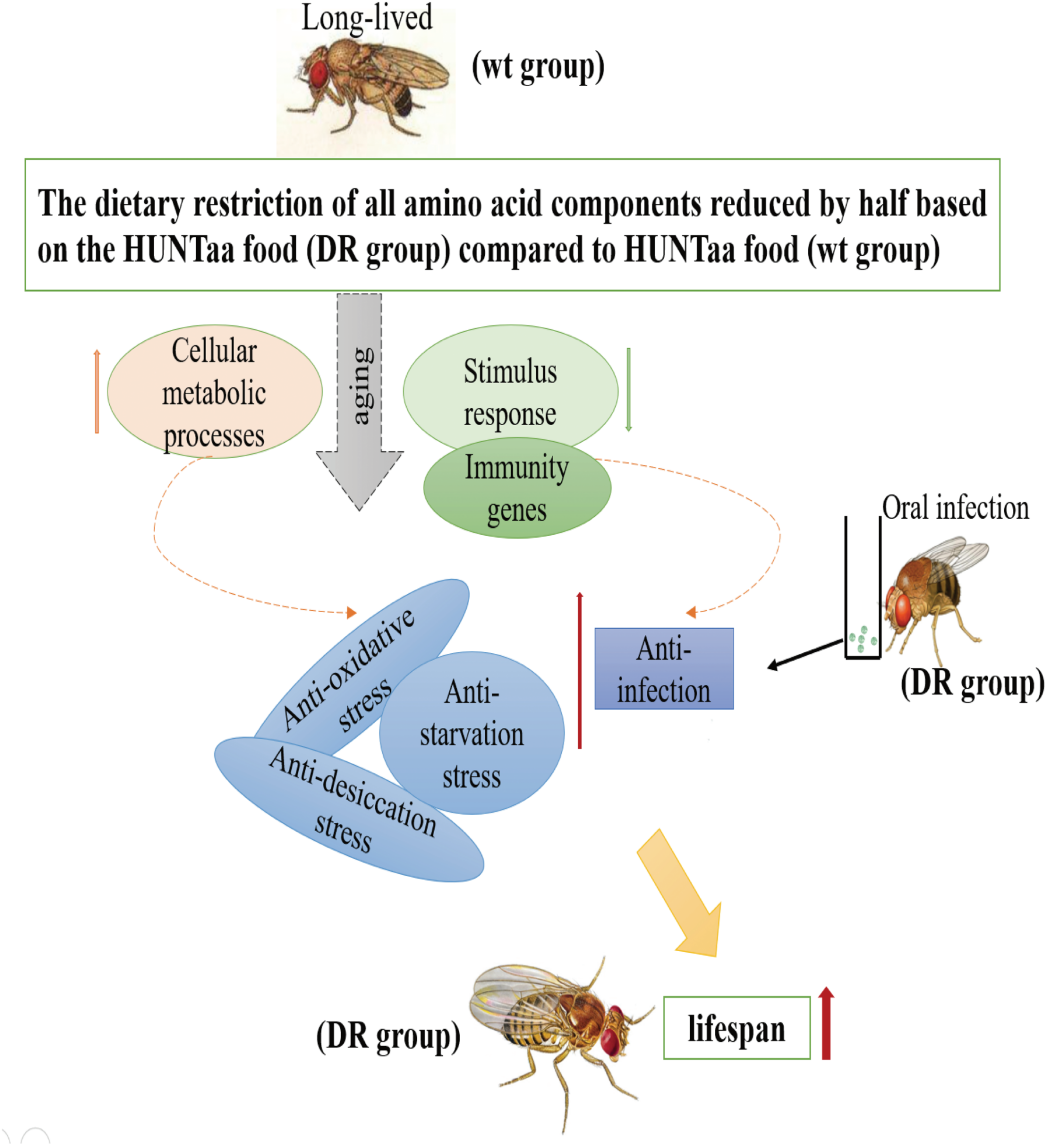
Summary of the work.

In this study, we found that the dietary restriction by halving amino acid component in the HUNTaa diet improved resistance to oxidative stress, desiccation stress, starvation stress, and resistance to bacterial infection, resulting in an extended *Drosophila* lifespan (Figure 6). Because the HUNTaa medium has been reported to already extend fly lifespan, our results suggest that health and lifespan-extending diet could be combined for additional benefit. Longitudinal transcriptomic analyses through aging process found that genes associated with FoxO pathway and cellular metabolism in *Drosophila* decreased with age, whereas genes associated with stimulus response and amino acid metabolism increased with age in flies raised in HUNTaa medium. On top of this, halving amino acids upregulated cellular metabolic processes and downregulated stimulus response and immune process genes during aging in these flies.

Dietary restriction is known to downregulate the mTOR pathway, glucose metabolism, lipid metabolism, and amino acid metabolism (15,16), and deficiencies of specific essential amino acids drive remodeling and programming of metabolic tissues to promote health (32). These pathways increased when flies were raised on HUNTaa (wt group) (Figure 2A and B), but halving amino acids in this diet (DR group) reversed the increase (Figure 2C and D), suggesting that the dietary restriction had similar effects on metabolic pathways as did other methods of dietary restrictions.

As our analysis revealed, the top 1 000 high-expressed genes and the bottom 1 000 low-expressed genes in the transcriptome expression at different time points of the 2 groups, and found that 88.9% of the high-expressed gene clusters in the 60-day-old DR group were identical to those in the 40-day-old wt group, and 89.2% of the high-expressed gene clusters in the 80-day-old DR group were identical to those in the 60-day-old wt group; whereas the percentage of identical low-expressed gene clusters were 33.7% and 34.5%, respectively. (Supplementary Figure 4) The delayed expression of overlapping genes is consistent with the fact that our dietary restriction treatment slowed the aging process in these flies.

The increase in inflammation during aging in *Drosophila* is manifested by increased expression of antimicrobial peptide genes (31,33). Dietary restriction can reduce inflammation, but the detailed mechanism is not fully understood (34). Dietary restriction extends lifespan by modulating a conserved innate immunity pathway that is regulated by p38 signaling and the transcription factor ATF-7 in *C. elegans* (35). However, our data showed that halving amino acid component of the HUNTaa medium slowed the aging of the immune system (Figure 5), but apparently through different pathways. We observed upregulated in rRNA metabolism, ribosomal biological processes and RNA transcription, and downregulated in the immune pathway at all 3 time points compared to wt group (Figure 3). But we did not observe any effects of our treatment on the p38-ATF-7 pathway. It is possible that this effect on immunity does not involve changes in the p38-ATF-7 pathway. It is also possible that other factors including diet, bacterial species, and different stages and methods of infection might have contributed to this difference.

Diet ingredients and proportions have an important impact on the reproducibility of healthy lifespan phenotypes in *Drosophila*. Our chemical medium based on well-defined ingredients as the *Drosophila* diet improved the reproducibility of the experiment, but, at the same time, may have led to differing observations from other studies. The infection protocol (of feeding aging flies also differs from other studies, where young flies were infected by injection) may also lead to the difference in pathways observed. Nevertheless, the increases in rRNA metabolism, ribosomal biological processes, and RNA transcription suggest a possible link with immunity. Ribosomal translation arrest increases with age, leading to ribosomal quality control overload and nascent peptide aggregation, resulting in impaired protein homeostasis (36). It is possible that dietary restriction treatment slowed immune function decline by improving protein homeostasis. This is an interesting future research direction, which requires significant efforts to establish this possible but important causal link.

## Materials and Methods

### Drosophila Stocks

After the mating of *GS-Gal4* females flies (Genotype: P{da-GSGAL4.T}, gift from Sichuan Agricultural University) and 03189 males flies (Genotype: y1 v1; P{TRiP}attP40/CyO, purchased from National Institute of genetics no. HMC03189), the female with red eyes and straight wings in the F1 generation was used as the experimental *Drosophila* for this experiment.

### Husbandry and Lifespan Analysis

The larval and mating stages of all strains of *Drosophila* were fed standard cornmeal–yeast medium (agar 7.6 g/L H_2_O, soy flour 13.5 g/L H_2_O, corn flour 92.7 g/L H_2_O, malt flour 61.8 g/L H_2_O, yeast [Angel instant dry yeast; China, GB/T 20886.1] 22.9 g/L H_2_O, syrup 206.1 g/L H_2_O, and propionic acid 6.4 mL/L H_2_O).

During 2–30 days of age, both wt and DR groups flies were fed wt group food (28). From 30 days of age, the food of DR group changed to DR group food. Table 1 for wt and DR food recipes and information.

After hatching, using *Drosophila* for lifespan statistics with 10 flies/tube, at least 20 tubes per experimental condition. All stocks were maintained at 25°C and 60% humidity, 12-hour light, 12-hour dark cycle. The flies were transferred to fresh food every 2 days, during which the number of dead flies was recorded. All lifespan data were analyzed with the log-rank test.

### Health Test Assays

A total of 10 flies per tube were used for the test, with at least 20 tubes per experimental condition. For the desiccation test, the flies were placed in an empty tube without medium, and the number of dead flies was counted every 2 hours. For the H_2_O_2_ oxidative stress test, circular filter paper was placed at the bottom of the culture tube, 50 μL of 6% glucose solution containing 30% H_2_O_2_ was added, the flies were put into the tube, and the number of dead flies was counted every 2 hours (37). For the starvation test, flies were placed in tubes containing 1.5% agarose, just to provide moisture but without any other nutrients. The number of dead flies was counted every day (38). All survival data were analyzed with the log-rank test.

### Bacterial Stocks and Infection

*Serratia marcescens* and *E faecalis* were obtained from the Renjie Jiao Lab at the Hoffmann Institute of Immunology, Guangzhou Medical University*. Serratia marcescens* and *E faecalis* were amplified in Luria-Bertani (LB) broth medium at 37°C. The absorbance was detected using 600 nm.

In *Drosophila* infected experiment with *S marcescens* (or *E faecalis*), *Drosophila* in the wt group were fed 100 µl of *S marcescens* with OD_600_ = 25 (or 100 µl of *E faecalis* with OD_600_=50) and 100 µl of wt group diet without agar; *Drosophila* in the DR group were fed 100 µl of *S marcescens* with OD_600_ = 25 (or 100 µl of *E faecalis* with OD_600_=50) and 100 µl of DR group diet without agar. The experimental method of bacterial load per *Drosophila* after infection was adopted from the 2018 *Journal of Visualized of Experiments* article (Oral Bacterial Infection and Shedding in *Drosophila melanogaster*) (39). All survival statistics were analyzed using log-rank test.

### qPCR (Quantitative Rea-ltime-PCR)

RNA was extracted with RNAiso Plus (TaKaRa, Kusatsu, Japan, RNAA00250); then, RNA was reverse transcribed into cDNA with Prime Script TMRT reagent kit with gDNA Eraser (TaKaRa, RR047Q), and qRT-PCR was performed using the Fast Start Universal SYBR Green Master (ROX) (Roche, Basel, Switzerland, 04913850001) and gene-specific primers. All the above experimental operations were performed according to the manufacturer’s procedures. Gene expression levels were calculated using the comparative C_T_ method. All primers used are listed in (Supplementary Table 2).

### RNA Isolation and Sequencing

Total RNA samples were extracted using the TRIzol method (Invitrogen). The polyA-enriched RNA-sequencing (RNA-seq) libraries were prepared for sequencing using the Illumina HiSeq 6000 platform.

### Bioinformatics Analysis

FastQC v0.11.9 (https://github.com/s-andrews/FastQC) was used for quality control of all raw data. The clean data were then aligned to the *D melanogaster* genome in UCSC (dm6) using Hisat2 v2.2.1 (40). The FeatureCounts (41) was used to count reads of genes. The raw count matrix was normalized as Transcripts Per Million.

DESeq2 (42) and limma (43) for DR and wt groups for differential gene screening in R. Differentially expressed genes and clusters 1–8 were subjected to GO enrichment analysis using the ClusterProfiler v4.2.1 package (44) and visualized using the “ggplot2” package in R. Functional enrichment analysis of Group A and Group B was conducted using Metascape (45) (https://metascape.org/gp/index.html#/main/step1). Significance was determined a priori at a Benjamini–Hochberg (BH)-corrected *p* value of < .05.

The gene clustering based on the log_2_FoldChange values of differential genes was referenced from *Analysis of myocardial cellular gene expression during pressure overload reveals matrix based functional intercellular communication* (46).

The gene expression time-series data were clustered using the fuzzy c-means algorithm in the Mfuzz package (47,48) of the R platform, and genes with consistent expression changes were grouped into Group A and Group B.

## Supplementary Material

glad225_suppl_Supplementary_Tables_1-2
