# Supplementary material for: Protein Restriction Effects on Healthspan and Lifespan in Drosophila melanogaster Are Additive With a Longevity-Promoting Diet
Source: J Gerontol A Biol Sci Med Sci. 2023 Sep 22;78(12):2251–9. doi: 10.1093/gerona/glad225 (PMC10692430; doi:10.1093/gerona/glad225)
Supplement: glad225_suppl_Supplementary_Tables_1-2 [file glad225_suppl_supplementary_tables_1-2.pdf]

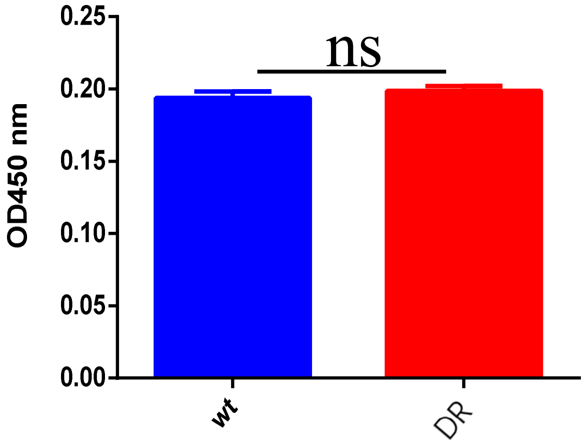


**Supplementary Figure 1. *Drosophila* feeding assay in wt and DR food.**

n=3, two-tailed t-test, p>0.05 (ns).

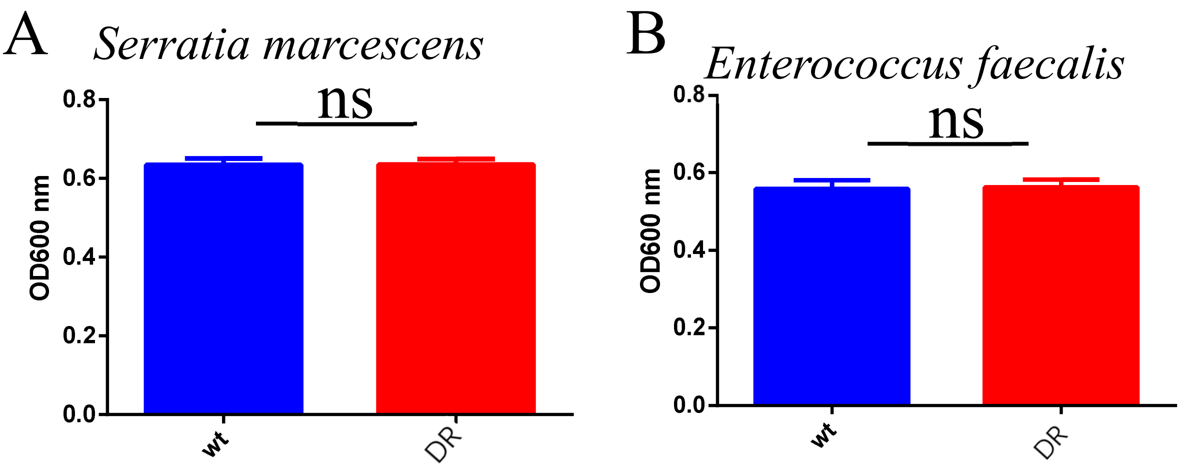


**Supplementary Figure 2. Proliferation test of bacteria in wt and DR food groups for 24h.**

(A) Proliferation test of *Serratia marcescens* in wt and DR group food for 24h, n=3, t-test, p>0.05 (ns).

(B) Proliferation test of *Enterococcus faecalis* in wt and DR group food for 24h, n=3, t-test, p>0.05 (ns).

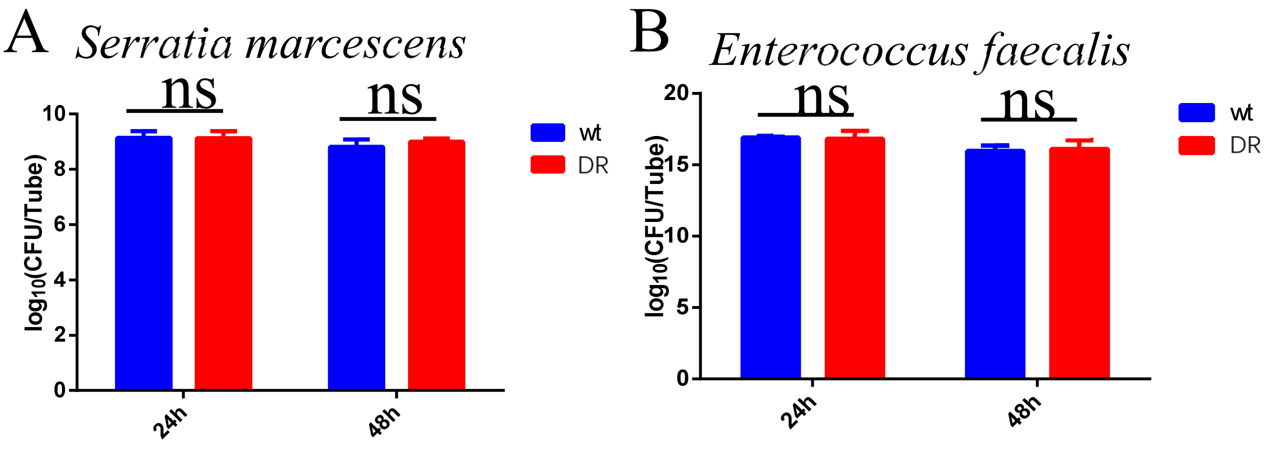


**Supplementary Figure 3. Determination of CFU/Tube after 24h and 48h of bacterial infection in wt and DR groups.**

(A) Determination of CFU/Tube after 24h and 48h of *Serratia marcescens* infection in wt and DR groups, n=3, two-tailed t-test, 24h p>0.05(ns), 48h p>0.05(ns).

(B) Determination of CFU/Tube after 24h and 48h of *Enterococcus faecalis* infection in wt and DR groups, n=3, two-tailed t-test, 24h p>0.05(ns), 48h p>0.05(ns).

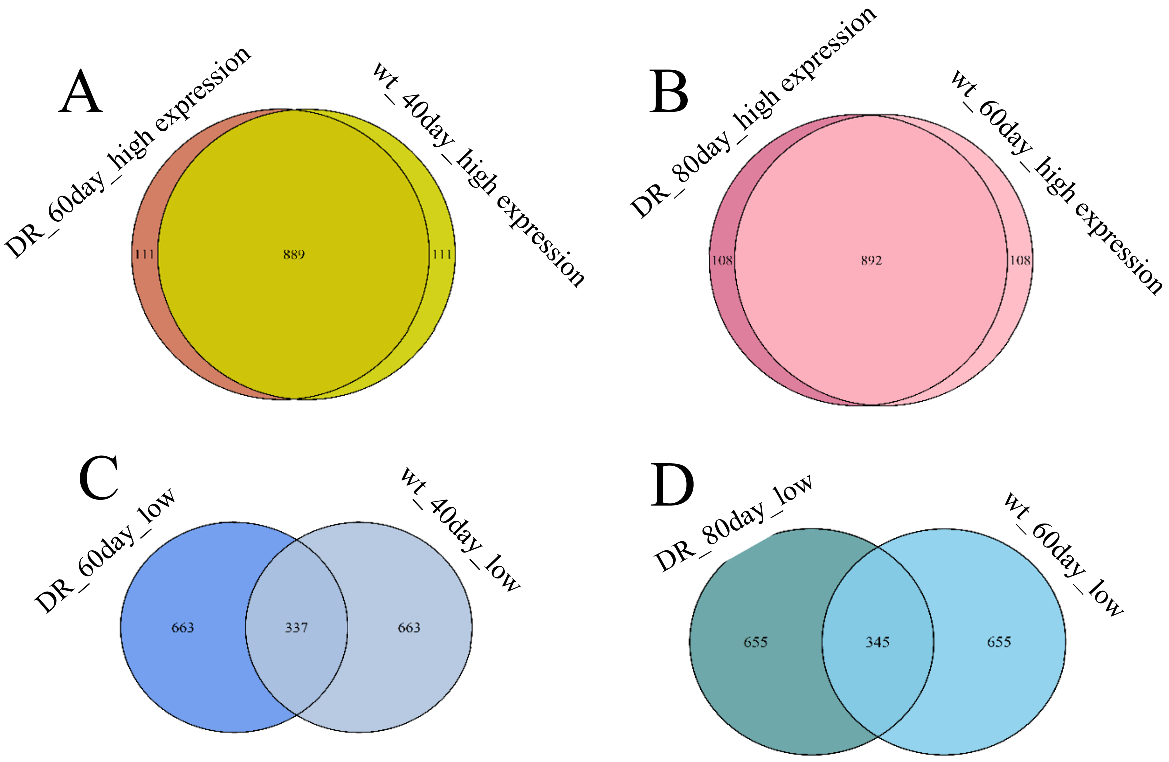

**Supplementary Figure 4. Intersection of the first 1000 highly expressed genes and the last 1000 low expressed genes in the transcriptome expression at different time points in DR and wt groups.**

(A) Intersection of the top 1000 highly expressed genes in the transcriptome data of the DR group at 60 days of age and the wt group at 40 days of age, 889 of these genes overlapped.

(B) Intersection of the top 1000 highly expressed genes in the transcriptome data of the DR group at 80 days of age and the wt group at 60 days of age, 892 of these genes overlapped.

(C) Intersection of the top 1000 low expressed genes in the transcriptome data of the DR group at 60 days of age and the wt group at 40 days of age, 337 of these genes overlapped.

(D) Intersection of the top 1000 low expressed genes in the transcriptome data of the DR group at 60 days of age and the wt group at 40 days of age, 345 of these genes overlapped.

**Supplementary Table 1**

| Gene | original_id | GroupA | GroupB | Biological Process (GO) |
|---|---|---|---|---|
| 34019 | CG15818 | 0 | 1 | GO:0044419 biological process involved in intersp organisms;GO:0008150 biological_process |
| 38717 | D19B | 1 | 0 | GO:0006355 regulation of DNA-templated transcript nucleic acid-templated transcription;GO:2001141 r process |
| 34800 | CG7916 | 0 | 1 | |
| 38075 | Reg-2 | 0 | 1 | |
| 36489 | Ser8 | 0 | 1 | GO:0006508 proteolysis;GO:0019538 protein metabol organonitrogen compound metabolic process |
| 318701 | CG31380 | 0 | 1 | |
| 326132 | CG31300 | 0 | 1 | GO:0071312 cellular response to alkaloid;GO:00713 caffeine;GO:0071415 cellular response to purine-c |

| | | | | |
|---|---|---|---|---|
| 39511 | CG10140 | 0 | 1 | |
| 33112 | peng | 1 | 0 | GO:0007475 apposition of dorsal and ventral imagi surfaces;GO:0008587 imaginal disc-derived wing ma regulation of translation |
| 42654 | CG33093 | 0 | 1 | |
| 39738 | CG10516 | 0 | 1 | GO:0043161 proteasome-mediated ubiquitin-dependen process;GO:0010498 proteasomal protein catabolic dependent protein catabolic process |
| 246416 | CG30049 | 0 | 1 | GO:0006508 proteolysis;GO:0019538 protein metabol organonitrogen compound metabolic process |
| 37754 | Alg3 | 1 | 0 | GO:0001960 negative regulation of cytokine-mediat pathway;GO:0010804 negative regulation of tumor n signaling pathway;GO:0060761 negative regulation stimulus |
| 38622 | Lkr | 0 | 1 | GO:2000252 negative regulation of feeding behavio regulation of cytosolic calcium ion concentration regulation of behavior |
| 37742 | St1 | 0 | 1 | GO:0051923 sulfation;GO:0006805 xenobiotic metabo cellular response to xenobiotic stimulus |
| 34167 | emb | 1 | 0 | GO:1900037 regulation of cellular response to hyp small subunit export from nucleus;GO:0046931 pore |
| 37097 | CG18540 | 0 | 1 | GO:0008150 biological_process |
| 38961 | Cbl | 1 | 0 | GO:0032353 negative regulation of hormone biosynt negative regulation of epidermal growth factor-ac activity;GO:0007176 regulation of epidermal growt activity |
| 36249 | Tret1-2 | 0 | 1 | GO:0055085 transmembrane transport;GO:0006810 tra establishment of localization |
| 30982 | sc | 0 | 1 | GO:0007540 sex determination, establishment of X: tubule tip cell differentiation;GO:0002067 glandu differentiation |
| 37095 | CG18538 | 0 | 1 | GO:0008150 biological_process |
| 32128 | CG9360 | 0 | 1 | |
| 41711 | CCHa1 | 0 | 1 | GO:0007218 neuropeptide signaling pathway;GO:0007 receptor signaling pathway;GO:0007165 signal tran |
| 42294 | Cyp12a4 | 0 | 1 | GO:0017085 response to insecticide;GO:0009636 res substance;GO:0042221 response to chemical |
| 19835472 | lncRNA:CR45426 | 0 | 1 | |
| 36932 | Amy-d | 0 | 1 | GO:0005975 carbohydrate metabolic process;GO:0044 process;GO:0071704 organic substance metabolic pr |
| 39064 | PGRP-LF | 0 | 1 | GO:0019731 antibacterial humoral response;GO:0061 peptidoglycan recognition protein signaling pathw humoral response |

| | | | | |
|---|---|---|---|---|
| 35955 | Drep2 | 1 | 0 | GO:1901215 negative regulation of neuron death;GO death;GO:0060548 negative regulation of cell deat |
| 12798579 | CG43074 | 1 | 0 | |
| 36569 | ReepB | 0 | 1 | GO:0007029 endoplasmic reticulum organization;GO: intracellular transport;GO:0060341 regulation of |
| 318225 | CG32816 | 1 | 0 | GO:0008150 biological_process |
| 42659 | CG33110 | 0 | 1 | GO:0019367 fatty acid elongation, saturated fatty elongation, unsaturated fatty acid;GO:0034625 fat monounsaturated fatty acid |
| 37488 | CG4363 | 0 | 1 | |
| 326116 | tau | 0 | 1 | GO:0061541 rhabdomere morphogenesis;GO:0008594 ph morphogenesis;GO:1901215 negative regulation of n |
| 33439 | Cyp309a2 | 0 | 1 | |
| 41686 | CG9312 | 0 | 1 | |
| 317954 | Drsl4 | 1 | 0 | GO:0050832 defense response to fungus;GO:0009620 defense response to other organism |
| 42783 | CG10184 | 0 | 1 | GO:0006545 glycine biosynthetic process;GO:000656 process;GO:0006567 threonine catabolic process |
| 42968 | CG7016 | 0 | 1 | GO:0008150 biological_process |
| 35042 | CLIP-190 | 0 | 1 | GO:0007349 cellularization;GO:0048646 anatomical in morphogenesis;GO:0009653 anatomical structure |
| 42701 | Nepl15 | 0 | 1 | GO:0016485 protein processing;GO:0051604 protein compound eye morphogenesis |
| 31527 | CG6041 | 0 | 1 | GO:0006508 proteolysis;GO:0019538 protein metabol organonitrogen compound metabolic process |
| 38858 | PGRP-SD | 0 | 1 | GO:0032499 detection of peptidoglycan;GO:0032490 bacterial origin;GO:0098581 detection of external |
| 39085 | aay | 0 | 1 | GO:0006564 L-serine biosynthetic process;GO:00065 process;GO:0009070 serine family amino acid biosy |
| 37309 | CG11192 | 0 | 1 | GO:0006508 proteolysis;GO:0019538 protein metabol organonitrogen compound metabolic process |
| 33127 | Cyp6t1 | 1 | 0 | GO:0009407 toxin catabolic process;GO:0042178 xen process;GO:0046701 insecticide catabolic process |
| 37158 | CG18609 | 0 | 1 | GO:0019367 fatty acid elongation, saturated fatty elongation, unsaturated fatty acid;GO:0034625 fat monounsaturated fatty acid |
| 40145 | Oat | 0 | 1 | GO:0010121 arginine catabolic process to proline arginine catabolic process to proline;GO:0019544 glutamate |
| 37069 | Pcl | 1 | 0 | GO:0050832 defense response to fungus;GO:0009620 ventral cord development |
| 40957 | CG7900 | 0 | 1 | GO:0009062 fatty acid catabolic process;GO:007232 catabolic process;GO:0044242 cellular lipid catab |

| | | | | |
|---|---|---|---|---|
| 31020 | CG5254 | 0 | 1 | GO:0015742 alpha-ketoglutarate transport;GO:19905 ketoglutarate transmembrane transport;GO:0006835 |
| 39485 | Syx13 | 0 | 1 | GO:0016081 synaptic vesicle docking;GO:0006904 ve exocytosis;GO:0048278 vesicle docking |
| 35344 | CG9328 | 1 | 0 | GO:0001558 regulation of cell growth;GO:0040008 r growth;GO:0051128 regulation of cellular componen |
| 37313 | GNBP-like3 | 0 | 1 | GO:0009595 detection of biotic stimulus;GO:000962 fungus;GO:0051606 detection of stimulus |
| 41777 | CG3505 | 0 | 1 | GO:0006508 proteolysis;GO:0019538 protein metabol organonitrogen compound metabolic process |
| 3885655 | CG34040 | 0 | 1 | GO:0008150 biological_process |
| 42456 | CG31199 | 0 | 1 | GO:0006508 proteolysis;GO:0019538 protein metabol organonitrogen compound metabolic process |
| 40232 | polo | 1 | 0 | GO:0030954 astral microtubule nucleation;GO:00486 commitment;GO:0051257 meiotic spindle midzone ass |
| 42051 | AdSL | 0 | 1 | GO:0044208 'de novo' AMP biosynthetic process;GO: process;GO:0046033 AMP metabolic process |
| 39870 | PGRP-SB1 | 0 | 1 | GO:0000270 peptidoglycan metabolic process;GO:000 process;GO:0006027 glycosaminoglycan catabolic pr |
| 38486 | CG14990 | 0 | 1 | GO:0006508 proteolysis;GO:0019538 protein metabol organonitrogen compound metabolic process |
| 38462 | CG12766 | 0 | 1 | |
| 33966 | nop5 | 1 | 0 | GO:0006364 rRNA processing;GO:0016072 rRNA metabo ribosome biogenesis |
| 35948 | Cyp4p3 | 0 | 1 | |
| 32392 | CG9411 | 0 | 1 | |
| 37487 | CG13492 | 0 | 1 | |
| 246663 | Prx2540-1 | 0 | 1 | GO:0042744 hydrogen peroxide catabolic process;GO detoxification;GO:0042743 hydrogen peroxide metab |
| 41791 | CG14857 | 0 | 1 | GO:0055085 transmembrane transport;GO:0006810 tra establishment of localization |
| 41087 | Rel | 0 | 1 | GO:0045428 regulation of nitric oxide biosyntheti regulation of nitric oxide biosynthetic process;G of nitric oxide metabolic process |
| 37572 | gas | 0 | 1 | |
| 46717 | arg | 0 | 1 | GO:0019547 arginine catabolic process to ornithin catabolic process;GO:0006591 ornithine metabolic |
| 34993 | grp | 1 | 0 | GO:0007348 regulation of syncytial blastoderm mit mitotic DNA replication checkpoint signaling;GO:0 cell cycle, embryonic |
| 35989 | CG1827 | 0 | 1 | GO:0006517 protein deglycosylation;GO:0009100 gly process;GO:1901135 carbohydrate derivative metabo |
| 38328 | CG14949 | 0 | 1 | |

| | | | | |
|---|---|---|---|---|
| 43463 | CG11951 | 0 | 1 | GO:0043171 peptide catabolic process;GO:1901565 o catabolic process;GO:0044248 cellular catabolic p |
| 36724 | CG12963 | 0 | 1 | |
| 35862 | PGRP-SC2 | 0 | 1 | GO:0000270 peptidoglycan metabolic process;GO:000 process;GO:0002814 negative regulation of biosynt antibacterial peptides active against Gram-negati |
| 47998 | rin | 1 | 0 | GO:0034063 stress granule assembly;GO:0008069 dor specification, ovarian follicular epithelium;GO:1 starvation |
| 47253 | dmGlut | 1 | 0 | GO:0044341 sodium-dependent phosphate transport;G of mitochondrial fusion;GO:0051938 L-glutamate im |
| 33649 | Elp3 | 1 | 0 | GO:0002926 tRNA wobble base 5-methoxycarbonylmeth thiouridinylation;GO:2000289 regulation of photor guidance;GO:0048789 cytoskeletal matrix organizat |
| 37489 | CG4377 | 0 | 1 | |
| 33615 | CG15422 | 0 | 1 | |
| 37086 | CG14499 | 0 | 1 | GO:0044419 biological process involved in intersp organisms;GO:0008150 biological_process |
| 41801 | CycC | 1 | 0 | GO:0045498 sex comb development;GO:0034472 snRNA snRNA processing |
| 42267 | Xrp1 | 0 | 1 | GO:0008285 negative regulation of cell population double-strand break repair;GO:0006281 DNA repair |
| 32663 | CG9609 | 1 | 0 | GO:0006357 regulation of transcription by RNA pol regulation of DNA-templated transcription;GO:1903 acid-templated transcription |
| 42008 | Decay | 0 | 1 | GO:0006915 apoptotic process;GO:0012501 programme death |
| 192535 | CG32032 | 0 | 1 | GO:0008150 biological_process |
| 37455 | Egfr | 0 | 1 | GO:0008071 maternal determination of dorsal/ventr epithelium, soma encoded;GO:0035310 notum cell fa photoreceptor cell fate determination |
| 35386 | CG9246 | 1 | 0 | GO:0035067 negative regulation of histone acetyla regulation of protein acetylation;GO:2000757 nega lysine acetylation |
| 41272 | Cyp12e1 | 0 | 1 | |
| 40282 | CG4858 | 0 | 1 | GO:0016226 iron-sulfur cluster assembly;GO:003116 assembly;GO:0006790 sulfur compound metabolic pro |
| 38363 | CG32485 | 0 | 1 | GO:0015914 phospholipid transport;GO:0015748 orga transport;GO:0006869 lipid transport |
| 35285 | AANATL3 | 0 | 1 | |
| 36692 | scb | 0 | 1 | GO:0003344 pericardium morphogenesis;GO:0060039 p development;GO:0007508 larval heart development |
| 31489 | CG3149 | 0 | 1 | GO:0034203 glycolipid translocation;GO:0046836 gl dolichol-linked oligosaccharide biosynthetic proc |

| | | | | |
|---|---|---|---|---|
| 41173 | p23 | 1 | 0 | GO:0051131 chaperone-mediated protein complex ass assembly;GO:0022618 ribonucleoprotein complex ass |
| 39020 | GNBP3 | 0 | 1 | GO:0002238 response to molecule of fungal origin; molecule of fungal origin;GO:0098581 detection of |
| 36333 | CG17739 | 0 | 1 | GO:0007155 cell adhesion;GO:0009987 cellular proc biological_process |
| 59149 | CG18765 | 0 | 1 | |
| 32833 | CG15046 | 0 | 1 | |
| 34554 | CG6287 | 0 | 1 | |
| 32907 | CG7914 | 1 | 0 | |
| 44790 | TwdlT | 0 | 1 | GO:0010171 body morphogenesis;GO:0040003 chitin-b development;GO:0042335 cuticle development |
| 37002 | Oxp | 1 | 0 | GO:0000122 negative regulation of transcription b II;GO:0051276 chromosome organization;GO:0045892 templated transcription |
| 36101 | CG12896 | 0 | 1 | GO:0098869 cellular oxidant detoxification;GO:009 toxic substance;GO:1990748 cellular detoxificatio |
| 35788 | Pabp2 | 1 | 0 | GO:0010560 positive regulation of glycoprotein bi positive regulation of protein glycosylation;GO:1 glycoprotein metabolic process |
| 31672 | CheA7a | 0 | 1 | GO:0007606 sensory perception of chemical stimulu perception;GO:0050877 nervous system process |
| 40137 | CG9372 | 0 | 1 | GO:0009620 response to fungus;GO:0009607 response stimulus;GO:0043207 response to external biotic s |
| 31738 | Smox | 1 | 0 | GO:0090256 regulation of cell proliferation invol wing morphogenesis;GO:0048580 regulation of post- development;GO:0032924 activin receptor signaling |
| 43054 | CG31104 | 0 | 1 | |
| 41728 | CG31326 | 0 | 1 | GO:0006508 proteolysis;GO:0019538 protein metabol organonitrogen compound metabolic process |
| 39997 | Wdr92 | 1 | 0 | GO:0033173 calcineurin-NFAT signaling cascade;GO: mediated signaling;GO:0097720 calcineurin-mediate |
| 39125 | CG18180 | 0 | 1 | GO:0006508 proteolysis;GO:0019538 protein metabol organonitrogen compound metabolic process |
| 42021 | CG17562 | 0 | 1 | GO:0035336 long-chain fatty-acyl-CoA metabolic pr CoA metabolic process;GO:1901568 fatty acid deriv |
| 38559 | Tie | 0 | 1 | GO:2000683 regulation of cellular response to X-r tyrosine phosphorylation;GO:0018212 peptidyl-tyro |
| 35824 | Mal-A1 | 0 | 1 | GO:0005975 carbohydrate metabolic process;GO:0044 process;GO:0071704 organic substance metabolic pr |
| 34824 | NimC4 | 0 | 1 | GO:0043652 engulfment of apoptotic cell;GO:000691 engulfment;GO:0043277 apoptotic cell clearance |

| | | | | |
|---|---|---|---|---|
| 41452 | CG18547 | 0 | 1 | GO:0070484 dehydro-D-arabinono-1,4-lactone metabo dehydro-D-arabinono-1,4-lactone biosynthetic proc metabolic process |
| 39390 | CAH2 | 0 | 1 | GO:0006730 one-carbon metabolic process;GO:004428 process;GO:0044237 cellular metabolic process |
| 37914 | CG4612 | 1 | 0 | GO:0045727 positive regulation of translation;GO: memory;GO:0034250 positive regulation of amide me |
| 37026 | Sardh | 0 | 1 | GO:1901053 sarcosine catabolic process;GO:1901052 process;GO:0033353 S-adenosylmethionine cycle |
| 3885601 | CG34054 | 0 | 1 | GO:0008150 biological_process |
| 39319 | CG11658 | 0 | 1 | GO:0031146 SCF-dependent proteasomal ubiquitin-de process;GO:0016567 protein ubiquitination;GO:0043 ubiquitin-dependent protein catabolic process |
| 41639 | PK2-R1 | 0 | 1 | GO:0007218 neuropeptide signaling pathway;GO:0007 receptor signaling pathway;GO:0007165 signal tran |
| 48338 | GstD5 | 0 | 1 | GO:0006749 glutathione metabolic process;GO:00065 acid metabolic process;GO:0006790 sulfur compound |
| 37748 | angel | 1 | 0 | GO:0008150 biological_process |
| 43431 | CG11842 | 0 | 1 | GO:0034976 response to endoplasmic reticulum stre response to stress;GO:0006508 proteolysis |
| 38579 | CG7509 | 0 | 1 | |
| 47895 | Nmdmc | 0 | 1 | GO:0035999 tetrahydrofolate interconversion;GO:00 metabolic process;GO:0006730 one-carbon metabolic |
| 48335 | GstD2 | 0 | 1 | GO:0006749 glutathione metabolic process;GO:00065 acid metabolic process;GO:0006790 sulfur compound |
| 32169 | Usp7 | 1 | 0 | GO:0031453 positive regulation of heterochromatin positive regulation of heterochromatin organizati regulation of chromatin organization |
| 2768715 | CheB42c | 0 | 1 | GO:0043695 detection of pheromone;GO:0019236 resp detection of chemical stimulus |
| 36857 | CG8306 | 0 | 1 | GO:0010025 wax biosynthetic process;GO:0010166 wa process;GO:0035336 long-chain fatty-acyl-CoA meta |
| 38884 | exex | 0 | 1 | GO:0060563 neuroepithelial cell differentiation;G differentiation;GO:0007412 axon target recognitio |
| 41642 | CheA87a | 0 | 1 | GO:0007606 sensory perception of chemical stimulu perception;GO:0050877 nervous system process |
| 318664 | CG31288 | 0 | 1 | |
| 35307 | Ugt37A1 | 1 | 0 | |
| 40947 | CG3223 | 1 | 0 | GO:0006511 ubiquitin-dependent protein catabolic modification-dependent protein catabolic process; dependent macromolecule catabolic process |
| 246444 | CG30083 | 0 | 1 | GO:0006508 proteolysis;GO:0019538 protein metabol organonitrogen compound metabolic process |

| | | | | |
|---|---|---|---|---|
| 35827 | Mal-A4 | 0 | 1 | GO:0005975 carbohydrate metabolic process;GO:0044 process;GO:0071704 organic substance metabolic pr |
| 36863 | Amyrel | 0 | 1 | GO:0005975 carbohydrate metabolic process;GO:0044 process;GO:0071704 organic substance metabolic pr |
| 19835061 | lncRNA:CR44832 | 0 | 1 | |
| 37052 | CG10910 | 0 | 1 | |
| 33635 | bdl | 0 | 1 | GO:0007158 neuron cell-cell adhesion;GO:0042065 g axon ensheathment |
| 39124 | CG18179 | 0 | 1 | GO:0006508 proteolysis;GO:0019538 protein metabol organonitrogen compound metabolic process |
| 3885606 | CG34043 | 0 | 1 | GO:0008150 biological_process |
| 246565 | Mal-A6 | 0 | 1 | GO:0005975 carbohydrate metabolic process;GO:0044 process;GO:0071704 organic substance metabolic pr |
| 38104 | CG13907 | 0 | 1 | GO:0015718 monocarboxylic acid transport;GO:00469 transport;GO:0015711 organic anion transport |
| 33074 | CG1304 | 0 | 1 | GO:0006508 proteolysis;GO:0019538 protein metabol organonitrogen compound metabolic process |
| 43483 | CG1907 | 0 | 1 | GO:0015743 malate transport;GO:0071423 malate tra transport;GO:0015729 oxaloacetate transport |
| 39395 | CG11529 | 0 | 1 | GO:0006508 proteolysis;GO:0019538 protein metabol organonitrogen compound metabolic process |
| 36273 | Zip48C | 0 | 1 | GO:0071577 zinc ion transmembrane transport;GO:00 transport;GO:0000041 transition metal ion transpo |
| 31732 | CG2260 | 1 | 0 | GO:0000462 maturation of SSU-rRNA from tricistron 5.8S rRNA, LSU-rRNA);GO:0030490 maturation of SSU small subunit biogenesis |
| 35826 | Mal-A3 | 0 | 1 | GO:0005975 carbohydrate metabolic process;GO:0044 process;GO:0071704 organic substance metabolic pr |
| 42808 | SdhD | 1 | 0 | GO:0006121 mitochondrial electron transport, succ ubiquinone;GO:0006099 tricarboxylic acid cycle;GO transport chain |
| 40278 | CG5059 | 0 | 1 | GO:0035794 positive regulation of mitochondrial m permeability;GO:0097345 mitochondrial outer membr permeabilization;GO:1902108 regulation of mitocho involved in apoptotic process |
| 33806 | obst-E | 0 | 1 | GO:0040003 chitin-based cuticle development;GO:00 development;GO:0007275 multicellular organism dev |
| 38679 | CG10477 | 0 | 1 | GO:0006508 proteolysis;GO:0019538 protein metabol organonitrogen compound metabolic process |
| 41727 | CG9649 | 0 | 1 | GO:0006508 proteolysis;GO:0019538 protein metabol organonitrogen compound metabolic process |
| 40009 | hid | 1 | 0 | GO:0009639 response to red or far red light;GO:00 light;GO:2000685 positive regulation of cellular |

| | | | | |
|---|---|---|---|---|
| 39066 | Pdxk | 0 | 1 | GO:0009443 pyridoxal 5'-phosphate salvage;GO:0042 process;GO:0042822 pyridoxal phosphate metabolic |
| 38623 | CG13285 | 0 | 1 | |
| 19835021 | lncRNA:CR45102 | 1 | 0 | |
| 33583 | odd | 0 | 1 | GO:0007366 periodic partitioning by pair rule gen derived leg joint morphogenesis;GO:0035285 append |
| 36636 | AttA | 0 | 1 | GO:0002213 defense response to insect;GO:0055093 hyperoxia;GO:0036296 response to increased oxygen |
| 43329 | HSPBAP1 | 1 | 0 | GO:0008150 biological_process |
| 38311 | Cyp4d20 | 0 | 1 | |
| 39463 | CG14120 | 0 | 1 | GO:0006309 apoptotic DNA fragmentation;GO:0006921 disassembly involved in execution phase of apopto nuclear changes |
| 33650 | MFS18 | 1 | 0 | GO:0055085 transmembrane transport;GO:0006810 tra establishment of localization |
| 43689 | CG1544 | 0 | 1 | GO:0006099 tricarboxylic acid cycle;GO:0009060 ae cellular respiration |
| 33425 | PIG-Wa | 1 | 0 | GO:0006506 GPI anchor biosynthetic process;GO:000 process;GO:0009247 glycolipid biosynthetic proces |
| 31933 | CG32694 | 0 | 1 | |
| 35407 | CG8665 | 0 | 1 | GO:0009256 10-formyltetrahydrofolate metabolic pr formyltetrahydrofolate catabolic process;GO:00093 compound catabolic process |
| 42791 | SPE | 0 | 1 | GO:0002816 regulation of biosynthetic process of against Gram-positive bacteria;GO:0006965 positiv process of antibacterial peptides active against bacteria;GO:0002804 positive regulation of antifu |
| 35174 | CG10470 | 1 | 0 | GO:0032469 endoplasmic reticulum calcium ion home calcium ion homeostasis;GO:0055074 calcium ion ho |
| 40059 | CG3902 | 0 | 1 | |
| 45577 | Faa | 0 | 1 | GO:1901999 homogentisate metabolic process;GO:190 process;GO:0006572 tyrosine catabolic process |
| 38129 | LysP | 0 | 1 | GO:0050829 defense response to Gram-negative bact response to bacterium;GO:0009617 response to bact |
| 41368 | CG6723 | 0 | 1 | GO:0006814 sodium ion transport;GO:0030001 metal monoatomic cation transport |
| 41337 | CG18577 | 0 | 1 | |
| 33885 | CG9498 | 0 | 1 | |
| 33686 | mxt | 1 | 0 | GO:1901190 regulation of formation of translation complex;GO:0036099 female germ-line stem cell pop maintenance;GO:0045727 positive regulation of tra |
| 36994 | CG6484 | 0 | 1 | GO:0008643 carbohydrate transport;GO:0055085 tran transport;GO:0071702 organic substance transport |

| | | | | |
|---|---|---|---|---|
| 38758 | CG10075 | 1 | 0 | GO:0017062 respiratory chain complex III assembly respiratory chain complex III assembly;GO:0017004 |
| 38490 | Chd64 | 0 | 1 | GO:0006939 smooth muscle contraction;GO:0035626 j signaling pathway;GO:0030522 intracellular recept |
| 49815 | Cht4 | 0 | 1 | GO:0006032 chitin catabolic process;GO:0046348 am process;GO:1901072 glucosamine-containing compoun |
| 40268 | CG5955 | 0 | 1 | GO:0006566 threonine metabolic process;GO:0006567 process;GO:0009068 aspartate family amino acid ca |
| 31252 | dyw | 0 | 1 | GO:0010840 regulation of circadian sleep/wake cyc positive regulation of circadian sleep/wake cycle positive regulation of circadian rhythm |
| 34802 | CG7968 | 0 | 1 | |
| 31921 | CG9686 | 0 | 1 | |
| 34145 | CG14274 | 0 | 1 | |
| 37232 | CalpA | 0 | 1 | GO:0016540 protein autoprocessing;GO:0000768 sync membrane fusion;GO:0006949 syncytium formation |
| 5740391 | CG34176 | 0 | 1 | |
| 35726 | CG12825 | 0 | 1 | |
| 43691 | CG1542 | 1 | 0 | GO:0042273 ribosomal large subunit biogenesis;GO: processing;GO:0016072 rRNA metabolic process |
| 32523 | mRpL3 | 1 | 0 | GO:0032543 mitochondrial translation;GO:0140053 m expression;GO:0006412 translation |
| 2768915 | CG33306 | 0 | 1 | GO:0008150 biological_process |
| 34903 | esg | 0 | 1 | GO:0007489 maintenance of imaginal histoblast dip regulation of DNA endoreduplication;GO:0001837 ep transition |
| 39825 | CG44836 | 0 | 1 | |
| 40540 | Nepl11 | 0 | 1 | GO:0016485 protein processing;GO:0051604 protein proteolysis |
| 10178859 | CG42728 | 0 | 1 | |
| 39530 | CG8745 | 0 | 1 | GO:0035094 response to nicotine;GO:0042221 respon response to stimulus |
| 46813 | CG6421 | 0 | 1 | GO:0050829 defense response to Gram-negative bact response to bacterium;GO:0009617 response to bact |
| 43067 | CHKov1 | 0 | 1 | |
| 37315 | CG13423 | 0 | 1 | GO:0043418 homocysteine catabolic process;GO:0000 catabolic process;GO:0050667 homocysteine metabol |
| 2768976 | CG32318 | 1 | 0 | |
| 41121 | CG8132 | 0 | 1 | GO:0006107 oxaloacetate metabolic process;GO:0006 process;GO:0006541 glutamine metabolic process |
| 32075 | CG1738 | 1 | 0 | GO:0008150 biological_process |
| 31726 | Ldsdh1 | 0 | 1 | |
| 41169 | Fst | 0 | 1 | GO:0009631 cold acclimation;GO:0009409 response t temperature stimulus |

| | | | | |
|---|---|---|---|---|
| 41174 | nmdyn-D7 | 1 | 0 | GO:0006183 GTP biosynthetic process;GO:0046039 GT process;GO:0006228 UTP biosynthetic process |
| 40210 | CG7298 | 0 | 1 | |
| 42524 | Rab1 | 0 | 1 | GO:0072741 protein localization to cell division localization to cleavage furrow;GO:0046597 negati into host cell |
| 41845 | ATPsynO | 1 | 0 | GO:0042776 proton motive force-driven mitochondri proton motive force-driven ATP synthesis;GO:00067 |
| 33163 | CG11374 | 0 | 1 | |
| 39507 | CG14105 | 0 | 1 | GO:0060271 cilium assembly;GO:0044782 cilium orga membrane bounded cell projection assembly |
| 38000 | CG3829 | 0 | 1 | |
| 36984 | cyp33 | 1 | 0 | GO:0000413 protein peptidyl-prolyl isomerization; modification;GO:0006457 protein folding |
| 42521 | e | 0 | 1 | GO:0043042 amino acid adenylylation by nonribosom synthase;GO:0048021 regulation of melanin biosynt negative regulation of melanin biosynthetic proce |
| 32072 | CG34348 | 1 | 0 | |
| 38264 | CG1139 | 0 | 1 | GO:0003333 amino acid transmembrane transport;GO: transport;GO:1903825 organic acid transmembrane t |
| 41596 | yellow-f2 | 0 | 1 | GO:0006583 melanin biosynthetic process from tyro biosynthetic process;GO:0042435 indole-containing process |
| 35940 | Rab32 | 0 | 1 | GO:0032438 melanosome organization;GO:0008057 eye organization;GO:0048072 compound eye pigmentation |
| 36356 | CG8834 | 0 | 1 | GO:0046949 fatty-acyl-CoA biosynthetic process;GO derivative biosynthetic process;GO:0001676 long-c process |
| 32170 | Cyp311a1 | 0 | 1 | |
| 326206 | CG11583 | 1 | 0 | GO:0000027 ribosomal large subunit assembly;GO:00 assembly;GO:0042273 ribosomal large subunit bioge |
| 46391 | CG11899 | 0 | 1 | GO:0006564 L-serine biosynthetic process;GO:00065 process;GO:0009070 serine family amino acid biosy |
| 42122 | AttD | 0 | 1 | GO:0050830 defense response to Gram-positive bact wounding;GO:0042742 defense response to bacterium |
| 32917 | CG7992 | 0 | 1 | |
| 38753 | Prat2 | 0 | 1 | GO:0009113 purine nucleobase biosynthetic process metabolic process;GO:0046112 nucleobase biosynthe |
| 42988 | mah | 0 | 1 | |
| 41125 | Task7 | 0 | 1 | GO:0030322 stabilization of membrane potential;GO transmembrane transport;GO:0006813 potassium ion |
| 41020 | Mkk4 | 0 | 1 | GO:0048082 regulation of adult chitin-containing pigmentation;GO:0033209 tumor necrosis factor-med pathway;GO:0048079 regulation of cuticle pigmenta |

| | | | | |
|---|---|---|---|---|
| 317887 | l(3)72Dp | 0 | 1 | GO:0046900 tetrahydrofolylpolyglutamate metabolic acid-containing compound metabolic process;GO:004 compound metabolic process |
| 31276 | CG14419 | 0 | 1 | |
| 41900 | CG5399 | 0 | 1 | GO:0008150 biological_process |
| 37519 | CG11073 | 0 | 1 | GO:0008150 biological_process |
| 32297 | CG1673 | 0 | 1 | GO:0009098 leucine biosynthetic process;GO:000909 process;GO:0006550 isoleucine catabolic process |
| 39111 | CG42673 | 0 | 1 | GO:0098974 postsynaptic actin cytoskeleton organi postsynaptic cytoskeleton organization;GO:0099173 |
| 326149 | CG31642 | 0 | 1 | GO:0099536 synaptic signaling;GO:0007267 cell-cel signaling |
| 31198 | Mct1 | 0 | 1 | GO:0015718 monocarboxylic acid transport;GO:00469 transport;GO:0015711 organic anion transport |
| 10178961 | CG42808 | 0 | 1 | |
| 31587 | Spat | 0 | 1 | GO:0019265 glycine biosynthetic process, by trans glyoxylate;GO:0009436 glyoxylate catabolic proces metabolic process |
| 43350 | mino | 0 | 1 | GO:0006072 glycerol-3-phosphate metabolic process metabolic process;GO:0034587 piRNA processing |
| 32108 | CG1561 | 0 | 1 | |
| 12798122 | CG43090 | 1 | 0 | GO:0008150 biological_process |
| 41520 | Cyp9f2 | 0 | 1 | GO:0035220 wing disc development;GO:0007444 imagi development;GO:0035295 tube development |
| 34799 | CG8997 | 0 | 1 | GO:0008150 biological_process |
| 41209 | FBXO11 | 1 | 0 | GO:0070922 RISC complex assembly;GO:1900368 regul gene silencing by RNA;GO:0060147 regulation of po silencing |
| 37413 | LSm1 | 1 | 0 | GO:0000290 deadenylation-dependent decapping of n mRNA;GO:0110156 methylguanosine-cap decapping;GO: |
| 39333 | CG10361 | 0 | 1 | GO:0006566 threonine metabolic process;GO:0006567 process;GO:0009068 aspartate family amino acid ca |
| 30986 | Cyp4g1 | 0 | 1 | GO:0006723 cuticle hydrocarbon biosynthetic proce biosynthetic process;GO:0120252 hydrocarbon metab |
| 43314 | CG5639 | 0 | 1 | GO:0007160 cell-matrix adhesion;GO:0031589 cell-s cell adhesion |
| 39554 | endos | 1 | 0 | GO:0032515 negative regulation of phosphoprotein activity;GO:0030104 water homeostasis;GO:0035308 protein dephosphorylation |
| 36186 | CG34227 | 0 | 1 | |
| 36047 | Def | 0 | 1 | GO:0019731 antibacterial humoral response;GO:0019 response;GO:0050830 defense response to Gram-posi |
| 32132 | CkIIbeta | 0 | 1 | GO:0008062 eclosion rhythm;GO:0007562 eclosion;GO protein serine/threonine phosphatase activity |

| | | | | |
|---|---|---|---|---|
| 34731 | l(2)k05911 | 0 | 1 | GO:0006508 proteolysis;GO:0019538 protein metabol organonitrogen compound metabolic process |
| 33591 | CG16713 | 0 | 1 | |
| 45574 | ida | 1 | 0 | GO:0070979 protein K11-linked ubiquitination;GO:0 mitotic metaphase/anaphase transition;GO:1901970 mitotic sister chromatid separation |
| 43268 | CG5880 | 1 | 0 | GO:0018345 protein palmitoylation;GO:0006497 prot lipoprotein biosynthetic process |
| 19835388 | lncRNA:CR44755 | 0 | 1 | |
| 40472 | CG11425 | 0 | 1 | GO:0046839 phospholipid dephosphorylation;GO:0030 modification;GO:0006644 phospholipid metabolic pr |
| 317900 | edin | 0 | 1 | GO:0050830 defense response to Gram-positive bact immune response;GO:0050829 defense response to Gr |
| 43936 | Men-b | 0 | 1 | GO:0006108 malate metabolic process;GO:0043648 di process;GO:0006090 pyruvate metabolic process |
| 33642 | Tps1 | 0 | 1 | GO:0070413 trehalose metabolism in response to st biosynthetic process;GO:0046351 disaccharide bios |
| 35080 | CG15153 | 0 | 1 | GO:0008150 biological_process |
| 42634 | mats | 1 | 0 | GO:0035329 hippo signaling;GO:0001934 positive re phosphorylation;GO:0042327 positive regulation of |
| 32905 | Mec2 | 0 | 1 | GO:0097205 renal filtration;GO:0097206 nephrocyte system process |
| 33756 | Cyp4ac3 | 0 | 1 | |
| 326270 | MtnD | 0 | 1 | GO:0046688 response to copper ion;GO:0010038 resp response to inorganic substance |
| 35258 | CG10366 | 1 | 0 | GO:0010468 regulation of gene expression;GO:00602 macromolecule metabolic process;GO:0019222 regula |
| 32444 | Cyp4s3 | 0 | 1 | |
| 43035 | CG11852 | 0 | 1 | GO:0032504 multicellular organism reproduction;GO reproduction;GO:0032501 multicellular organismal |
| 37059 | CG10912 | 0 | 1 | GO:0009631 cold acclimation;GO:0009409 response t temperature stimulus |
| 41314 | CG14695 | 0 | 1 | |
| 43599 | CecC | 0 | 1 | GO:0019731 antibacterial humoral response;GO:0019 response;GO:0050830 defense response to Gram-posi |
| 31958 | CG2909 | 0 | 1 | |
| 44008 | Hex-C | 0 | 1 | GO:0001678 cellular glucose homeostasis;GO:005115 metabolic process;GO:0046835 carbohydrate phospho |
| 43596 | CecA1 | 0 | 1 | GO:0002213 defense response to insect;GO:0019731 response;GO:0019730 antimicrobial humoral respons |
| 44183 | ScpX | 0 | 1 | GO:0032373 positive regulation of sterol transpor regulation of cholesterol transport;GO:0032377 re lipid transport |

| | | | | |
|---|---|---|---|---|
| 43598 | CecB | 0 | 1 | GO:0002213 defense response to insect;GO:0019731 response;GO:0019730 antimicrobial humoral respons |
| 32299 | GstT4 | 0 | 1 | GO:0006749 glutathione metabolic process;GO:00065 acid metabolic process;GO:0006790 sulfur compound |
| 39616 | CG17839 | 0 | 1 | |
| 32954 | CG14220 | 1 | 0 | GO:0016575 histone deacetylation;GO:0006476 prote protein deacylation |
| 41829 | Spn88Eb | 0 | 1 | GO:1990399 epithelium regeneration;GO:2000035 reg division;GO:0042246 tissue regeneration |
| 246581 | GstE9 | 0 | 1 | GO:0006749 glutathione metabolic process;GO:00065 acid metabolic process;GO:0006790 sulfur compound |
| 33524 | FASN1 | 0 | 1 | GO:0035356 cellular triglyceride homeostasis;GO:0 disaccharide stimulus;GO:0071329 cellular respons |
| 40048 | Cat | 0 | 1 | GO:0038001 paracrine signaling;GO:0007568 aging;G catabolic process |
| 326256 | CG33107 | 1 | 0 | |
| 41522 | CG5196 | 0 | 1 | GO:0018230 peptidyl-L-cysteine S-palmitoylation;G diacylglycerol-L-cysteine biosynthetic process fr cysteine;GO:0018198 peptidyl-cysteine modificatio |
| 41521 | Sccpdh2 | 0 | 1 | GO:0009247 glycolipid biosynthetic process;GO:000 process;GO:1903509 liposaccharide metabolic proce |
| 318582 | CG31076 | 0 | 1 | |
| 117369 | Desat1 | 0 | 1 | GO:0006723 cuticle hydrocarbon biosynthetic proce biosynthetic process;GO:0120252 hydrocarbon metab |
| 37785 | ken | 1 | 0 | GO:0045496 male analia development;GO:0045497 fem development;GO:0007487 analia development |
| 36248 | Tret1-1 | 0 | 1 | GO:0015771 trehalose transport;GO:0015766 disacch oligosaccharide transport |
| 38259 | mv | 0 | 1 | GO:0045771 negative regulation of autophagosome s autophagosome size;GO:0031339 negative regulation |
| 19835457 | IBIN | 0 | 1 | |
| 41894 | AOX1 | 0 | 1 | GO:0042817 pyridoxal metabolic process;GO:0042816 process;GO:0006767 water-soluble vitamin metaboli |
| 19834718 | sud1 | 1 | 0 | GO:0006449 regulation of translational terminatio hydroxylation;GO:0018126 protein hydroxylation |
| 34045 | Tep3 | 0 | 1 | GO:0050830 defense response to Gram-positive bact immune response;GO:0006955 immune response |
| 37566 | ppk12 | 0 | 1 | GO:0035725 sodium ion transmembrane transport;GO: transport;GO:0098662 inorganic cation transmembra |
| 50225 | Prosap | 1 | 0 | GO:0097107 postsynaptic density assembly;GO:00986 specialization assembly;GO:1904861 excitatory syn |
| 41729 | CG9631 | 0 | 1 | GO:0006508 proteolysis;GO:0019538 protein metabol organonitrogen compound metabolic process |

| | | | | |
|---|---|---|---|---|
| 318559 | spdo | 0 | 1 | GO:0045035 sensory organ precursor cell division; morphogenesis;GO:0045746 negative regulation of N |
| 32885 | CG7332 | 1 | 0 | GO:0071108 protein K48-linked deubiquitination;GO deubiquitination;GO:0070646 protein modification |
| 41457 | Ect3 | 0 | 1 | GO:0005975 carbohydrate metabolic process;GO:0044 process;GO:0071704 organic substance metabolic pr |
| 36484 | AttC | 0 | 1 | GO:0019731 antibacterial humoral response;GO:0019 response;GO:0050830 defense response to Gram-posi |
| 326235 | Cubn | 0 | 1 | GO:0070293 renal absorption;GO:0097017 renal prot renal filtration |
| 32915 | CG14196 | 0 | 1 | GO:0015718 monocarboxylic acid transport;GO:00469 transport;GO:0015711 organic anion transport |
| 36595 | Arc1 | 0 | 1 | GO:0110077 vesicle-mediated intercellular transpo response to starvation;GO:0010496 intercellular t |
| 36894 | CG6435 | 0 | 1 | |
| 37058 | CG10911 | 0 | 1 | GO:0008150 biological_process |
| 35241 | CG10137 | 0 | 1 | GO:0060271 cilium assembly;GO:0044782 cilium orga membrane bounded cell projection assembly |
| 42583 | Qsox2 | 0 | 1 | GO:0006457 protein folding;GO:0019953 sexual repr reproduction |
| 37028 | CG5009 | 0 | 1 | GO:0140493 very long-chain fatty acid beta-oxidat peroxide biosynthetic process;GO:1903409 reactive process |
| 32737 | stas | 0 | 1 | GO:0032224 positive regulation of synaptic transm cholinergic;GO:0032222 regulation of synaptic tra cholinergic;GO:0050806 positive regulation of syn |
| 38386 | CG14957 | 0 | 1 | |
| 34817 | Cyp28a5 | 0 | 1 | |
| 43659 | CG15546 | 0 | 1 | GO:0008150 biological_process |
| 36366 | Nepl10 | 0 | 1 | GO:0016485 protein processing;GO:0051604 protein proteolysis |
| 317894 | CG32170 | 0 | 1 | GO:0008150 biological_process |
| 36893 | CG6429 | 0 | 1 | GO:0050829 defense response to Gram-negative bact response to bacterium;GO:0009617 response to bact |
| 35629 | Tsp42Er | 0 | 1 | |
| 43597 | CecA2 | 0 | 1 | GO:0002213 defense response to insect;GO:0019731 response;GO:0019730 antimicrobial humoral respons |
| 33559 | CG2772 | 0 | 1 | GO:0006629 lipid metabolic process;GO:0044238 pri process;GO:0071704 organic substance metabolic pr |
| 40349 | ppl | 0 | 1 | GO:0019464 glycine decarboxylation via glycine cl glycine catabolic process;GO:0009249 protein lipo |
| 37183 | DptA | 0 | 1 | GO:0055093 response to hyperoxia;GO:0036296 respo levels;GO:0019731 antibacterial humoral response |

| | | | | |
|---|---|---|---|---|
| 37010 | swi2 | 0 | 1 | GO:0035073 pupariation;GO:0035210 prepupal develo ecdysone |
| 50323 | CG15210 | 0 | 1 | |
| 35616 | Tsp42Eg | 0 | 1 | |
| 326185 | CG32054 | 0 | 1 | GO:0008645 hexose transmembrane transport;GO:0015 transmembrane transport;GO:0034219 carbohydrate t |
| 36183 | Listericin | 0 | 1 | GO:0051607 defense response to virus;GO:0140546 d symbiont;GO:0050830 defense response to Gram-posi |
| 3885667 | CG42729 | 0 | 1 | |
| 34552 | hgo | 0 | 1 | GO:0006558 L-phenylalanine metabolic process;GO:0 catabolic process;GO:1902221 erythrose 4-phosphat amino acid metabolic process |
| 49808 | Spn42Dd | 0 | 1 | GO:1905034 regulation of antifungal innate immune regulation of antifungal innate immune response;G receptor ligand protein activation cascade |
| 318776 | CG31516 | 0 | 1 | GO:0008150 biological_process |
| 326234 | l(1)G0320 | 1 | 0 | |
| 31503 | CG3097 | 0 | 1 | GO:0006508 proteolysis;GO:0019538 protein metabol organonitrogen compound metabolic process |
| 5740871 | snoRNA:Psi18S-525k | 0 | 1 | |
| 326124 | CG31200 | 0 | 1 | GO:0006508 proteolysis;GO:0019538 protein metabol organonitrogen compound metabolic process |
| 40267 | mag | 0 | 1 | GO:0042632 cholesterol homeostasis;GO:0055092 ste triglyceride homeostasis |
| 33038 | Obp19b | 0 | 1 | GO:0050909 sensory perception of taste;GO:0007608 smell;GO:0007606 sensory perception of chemical s |
| 41679 | Adgf-D | 0 | 1 | GO:0006154 adenosine catabolic process;GO:0046085 process;GO:0046102 inosine metabolic process |
| 39135 | CG16711 | 1 | 0 | GO:0008150 biological_process |
| 40067 | CG6839 | 0 | 1 | GO:0006309 apoptotic DNA fragmentation;GO:0006921 disassembly involved in execution phase of apopto nuclear changes |
| 31701 | bys | 1 | 0 | GO:0006364 rRNA processing;GO:0016072 rRNA metabo ribosome biogenesis |
| 43330 | ALiX | 0 | 1 | GO:0097734 extracellular exosome biogenesis;GO:19 secretion;GO:0140112 extracellular vesicle biogen |
| 32147 | CG10352 | 0 | 1 | GO:0006470 protein dephosphorylation;GO:0016311 d phosphate-containing compound metabolic process |

**Supplementary Table 2**

| Primer name | Primer sequencing |
|---|---|
| AttA-F | CCCGGAGTGAAGGATG |
| AttA-R | GTTGCTGTGCGTCAAG |

| | |
|---|---|
| DptA-F | CGATGGTTTTGGCTTTGCAG |
| DptA-R | TCCTCCATTCAGTCCAATCTCG |
| mtd-F | ACCTCACAACATGGTTTCGC |
| mtd-R | AAGTGATCCGACACATGCAG |
| CeCA2-F | GTTTTCGTCGCTCTCATTCTGG |
| CeCA2-R | TGTTGAGCGATTCCCAGTCC |
| Tep3-F | GTCACCGCTTGTTTGCTTTG |
| Tep3-R | TGGCAGATTCAGGTGCATTG |
| DptB-F | TGCATTTCACCGCTAGTCTTC |
| DptB-R | CAGGCTGCAGATTCACAATCTC |
| Def-F | GCTCAGCCAGTTTCCGATGT |
| Def-R | TCCTGGTGGGCATCCTCAT |
| Toll7-F | ATCCATCGCAACCCAGTGG |
| Toll7-R | GCTGTCGCTCAATGAGACG |
| IM2-F | ACCGTCTTTGTGTTCGGTCT |
| IM2-R | TGCAGTCCCCGTTGATTACC |
| Drs-F | CGTGAGAACCTTTTCCAATATGATG |
| Drs-R | TCCCAGGACCACCAGCAT |
